# Making sense of (exceptional) causal relations. A cross-cultural and cross-linguistic study

**DOI:** 10.3389/fpsyg.2015.01645

**Published:** 2015-10-30

**Authors:** Olivier Le Guen, Jana Samland, Thomas Friedrich, Daniel Hanus, Penelope Brown

**Affiliations:** ^1^Linguistics, Centro de Investigaciones y Estudios Superiores en Antropologia SocialMexico City, Mexico; ^2^Georg-Elias-Müller-Institute of Psychology, University of GottingenGottingen, Germany; ^3^Institute of Social and Cultural Anthropology, University of HamburgHamburg, Germany; ^4^Developmental and Comparative Psychology, Max Planck Institute of Evolutionary AnthropologyLeipzig, Germany; ^5^Language Acquisition, Max Planck Institute for PsycholinguisticsNijmegen, Netherlands

**Keywords:** causality, chance, cross-cultural cognition, coincidence, intentionality

## Abstract

In order to make sense of the world, humans tend to see causation almost everywhere. Although most causal relations may seem straightforward, they are not always construed in the same way cross-culturally. In this study, we investigate concepts of “chance,” “coincidence,” or “randomness” that refer to assumed relations between intention, action, and outcome in situations, and we ask how people from different cultures make sense of such non-law-like connections. Based on a framework proposed by Alicke (2000), we administered a task that aims to be a neutral tool for investigating causal construals cross-culturally and cross-linguistically. Members of four different cultural groups, rural Mayan Yucatec and Tseltal speakers from Mexico and urban students from Mexico and Germany, were presented with a set of scenarios involving various types of causal and non-causal relations and were asked to explain the described events. Three links varied as to whether they were present or not in the scenarios: Intention-to-Action, Action-to-Outcome, and Intention-to-Outcome. Our results show that causality is recognized in all four cultural groups. However, how causality and especially non-law-like relations are interpreted depends on the type of links, the cultural background and the language used. In all three groups, Action-to-Outcome is the decisive link for recognizing causality. Despite the fact that the two Mayan groups share similar cultural backgrounds, they display different ideologies regarding concepts of non-law-like relations. The data suggests that the concept of “chance” is not universal, but seems to be an explanation that only some cultural groups draw on to make sense of specific situations. Of particular importance is the existence of linguistic concepts in each language that trigger ideas of causality in the responses from each cultural group.

## Introduction^1^

Humans see causality everywhere and in everything. Because the interpretation of causality is so omnipresent in everyday life, it is no surprise that it has been the subject of many studies (Shaver, 1895; Sperber et al., 1996, inter alia; Bender and Beller, 2011b; Bender et al., 2012). Interdisciplinary studies of causal thinking remain, however, rare in the social sciences^2^. Psychologists typically study physical and social causality in controlled laboratory settings, but seldom consider cross-cultural comparisons. Anthropologists, in contrast, are primarily interested in the cultural and cross-cultural study of concepts like “chance,” “witchcraft,” and “fate,” but seldom investigate these questions in a rigorously controlled manner, for example by using experimental tasks (for exceptions see Bloch, 1998; Tomasello et al., 2005; Astuti and Bloch, 2015). Linguists have looked systematically at how causality is encoded in the grammar of various languages (e.g., Wolff, 2003; Sanders and Sweetser, 2009; Sanders et al., 2009; Kwon, 2012), yet the cultural consequences of such variation are rarely discussed (exceptions include Evans, 2009; Bohnemeyer and Pederson, 2011; San Roque et al., 2012). It should be noted, however, that an interdisciplinary approach is increasingly common and has been shown to provide more comprehensive results in various domains, especially in cross-cultural studies (see for instance, Atran et al., 2002; Bang et al., 2007; Bender and Beller, 2011b). This paper is an outcome of an interdisciplinary research group that united, among others, psychologists, anthropologists and linguists to address the issue of causality from a cross-cultural perspective. Although our study is mainly exploratory, we believe it shows promising results for future cross-cultural comparisons of causal cognition.

In this paper, we explore how people in different cultural settings explain typical causation but also exceptional relations between events, such as non-law-like relations between cause and effect—what in English is referred to under labels such as “chance,” “coincidence,” or “luck.” One motivation for this study and for the chosen cultural groups lies in the fact that some languages seem to lack words for such expressions, as is the case in the Mayan languages in contrast with most Indo-European languages (like German or Spanish). The main questions behind this study are these: Do humans from different cultural groups have a similar understanding of causality? To what extent is causation or the absence of clear causal links interpreted in culturally specific ways? Do people in all cultures have a concept of “chance” or “coincidence” despite the fact that some might lack linguistic labels for such concepts? In order to try to answer these questions, we designed a verbal task that consists of various systematically varied scenarios which participants are asked to interpret. Although causal reasoning can be considered a basic cognitive process, language is crucial not merely to express causal relations but also, we argue, to codify them (hence to interpret causality in terms of categories of events).

In order to explore causation across cultures and avoid ethnocentricity, we chose not to start with a priori concepts like “chance” or “bad luck” for instance, but instead to use a logical combination of causal links so that our scenarios were structurally identical across cultures. We used the framework proposed by Alicke (2000) that was originally developed to examine aspects of blame attribution. The central idea is that causal relations are divided into separate links between intention, action, and outcome. As this segmentation allows for a more detailed analysis of the single causal components involved, it provided a good basis for designing a “neutral” tool to investigate causal cognition cross-culturally and cross-linguistically. Such a tool, which we present in more detail below, allows us to examine cultural patterns of the inferences people draw related to causality and how these are linguistically codified.

This tool was tested within four groups of different cultural backgrounds and languages. The four groups consist of German students from the university of Göttingen, Mexican Spanish-speaking students from the UNACH University (Chiapas, Mexico), and people from two indigenous Mexican groups: Yucatec Mayans from the Yucatec Peninsula and Tseltal Mayans from the highlands of Chiapas. Both groups of students (German and non-indigenous Mexican) have a high level of literacy and live in an urban environment, while members of both Mayan groups are in their great majority non-literate and live mainly a peasant lifestyle based on slash and burn agriculture.

The choice of these four groups was primarily motivated by the decision to compare groups from “western”^3^ cultures (i.e., WEIRD, western, educated, post-industrial, rich, developed, etc., see Henrich et al., 2010), the Germans and the Mexicans, with “non-western” (subsistence, rural, traditional) groups, the Mayans. In addition to their lifestyle, the groups differ linguistically: German and Spanish are Indo-European languages; Tseltal and Yucatec are Mayan languages. We also wanted to control for effects within the two language families, i.e., the German vs. the Mexican Spanish and the Tseltal Maya vs. the Yucatec Maya. Furthermore, the comparison between the Mexican Spanish group and the Mayans is interesting, since all three groups live in the same region but have quite different ways of life.

The two Mayan groups were chosen because they lack explicit words for “coincidence” or “chance” and, despite both groups having related cultural and linguistic backgrounds, they seem to have different ideas about non-law-like relations between events (as shown below). The German student group was chosen as a typical student sample from a western university. The Mexican Spanish student group was included to contrast with both, the German students and the Mayans. Mexican Spanish belongs to the Romance family and non-indigenous Mexicans do not share many of the Mayan cultural traits. However, Mayans and Mexicans live in the same country and have a different way of life from that of most European groups (like German or Iberian Spanish people).

### Causality and non-law-like relations between events

The notion of causality is omnipresent in science and in daily life and applies to physical events as well as to human (inter)actions. In the social domain with which we are concerned, judgments of causality are often related to judgments about responsibility (Sousa, 2009), blame (Shaver, 1895; Alicke, 2000), or intentionality (Searle, 1983). In this section, we propose some basic working definitions of what we will consider “causality” or “causation” and what we consider to be “(non-)law-like relations between events.” We consider causality to be the relationship between an event 1 (the *cause*) and an event 2 (the *effect*), where the second event is understood as a consequence or the outcome of the first. The issue of causality is far from unproblematic since causal reasoning is, for humans, generally based not so much on observable processes but on assumptions that arise by reason of observations between events or prior knowledge (see Lagnado et al., 2007). Sometimes the relation between two events is considered to be a causal one even without any known causal (physical) mechanism that links the one to the other; for example, in the social domain, where a person's frowning can cause another person to react. As Waldmann and Hagmayer point out, “the main question of how we distinguish causal relations from accidental sequences of events remains highly debated” (Waldmann and Hagmayer, 2001, p. 28), and this is the very reason for exploring how people from different cultural backgrounds do or do not make this distinction and how they differ in judging such sequences of events.

In the psychological literature about causal judgments (based on empirical studies that are typically conducted with undergraduates of “western” universities), statistical relations, temporal order, intervention and prior knowledge are known cues for causal structure, i.e., for the question whether a relation between two events is considered to be a causal one (Lagnado et al., 2007). However, it is known that there sometimes are cultural differences in causal attribution (Bender and Beller, 2011a, 2013) and it is thus possible that other factors influence the causal judgments that people who are from other cultural backgrounds than the “western” population make.

One interesting idea in this regard is that an agent's intentions or desires can cause things to happen—even without any physical connection (e.g., without being mediated by the agent's action). The influence of mental states like intentions on the occurrence of events is sometimes called “magical thinking” or “mental causation” and it has been claimed that it is more prevalent in some cultural groups than in others. In some cultures it is, for example, not uncommon to infer a causal relationship between somebody's thoughts about a snake and its appearance a few seconds later (see Ojalehto et al., 2013). Although there may be superstitious beliefs and magical thinking among the “western” population too (for instance if a soccer fan believes that his wearing a fan scarf will contribute to the chance of his team's win), psychological studies about causal judgments of “western” undergraduates consistently deal with *events*, such as actions or physical processes, negating the possibility that mental states can be considered as causes for events that become manifest in the physical world.

What is important to note, though, is that only a very low percentage of events that are contiguous in space and time are causally related to each other. For example, the pressing of the doorbell button might be causally related to the doorbell ringing, whereas the simultaneous scratching of one's nose probably is not. There are millions of events that happen more or less at the same time, but most of them are not recognized as being even possibly related to each other. When attention is driven toward two (or more) events that happen in direct sequence but are not known to be directly causally related, English speakers would use words such as “chance,” “coincidence,” or “(bad) luck,” in order to make sense of the temporal correspondence of these events. These words refer to events that are somehow related but leave some margin of interpretation, in contrast, for instance, with a direct causal formulation such as “I rang the doorbell” (which also is, in reality, an interpretation since the speaker might not know if there is indeed a causal relation between the pressing of the button and the bell ringing). We will refer to concepts like “chance,” “coincidence,” or “(bad) luck” as “non-law-like relationship explanations,” in contrast to direct causal explanations for events.

An interesting framework to account for the influence of “mechanical” connections between an action and an outcome, the use of non-law-like-relationship explanations and the influence of mental states on the attribution of causal relationships is provided by Alicke's *Culpable Control Model* (Alicke, 2000), which was developed to capture lay people's blame judgments. In this model, the causal impact of an agent's action on an outcome (i.e., Causal Control) is only one of three components of *personal control* which is crucial to blame and responsibility judgments. Next to this causal control link between action and outcome (A → O; also considered *behavior to consequence* by Alicke), blame evaluations are also based on volitional behavior control that is represented by the link from intention to action (I → A; or *mind to behavior*) and on volitional outcome control represented by the link from intention to outcome (I → O; or *mind to consequence*). Whereas the Intention-to-Action link determines whether an action was intended or not, the Intention-to-Outcome link defines the desire of the agent, i.e., whether (s)he foresaw and wanted the outcome event to happen. Personal control, which is crucial to blame judgments, is maximized if all three links are present: an agent who wanted the outcome to happen and who intentionally performed a certain action that caused the outcome is more blameworthy than an agent whose accidental action caused an outcome (s)he did not want (Alicke, 2000; Cushman, 2008). If a boy breaks his neighbor's window, for example, his action is usually considered to be more blameworthy when he wanted to destroy the window and intentionally kicked a ball in the direction of his neighbor's house compared to a situation in which he accidentally broke it, without wanting it or being able to foresee that his shot could lead to this damage.

Although evaluations of blame and responsibility will not be directly addressed in this study, we consider Alicke's structural linkages to serve as a neutral framework for our aim of investigating causal attribution and non-law-like relationship attributions in social contexts among people from different cultures. As mentioned earlier, we are especially interested in evaluating the extent to which participants consider intentionality to be relevant with regard to the realization of the outcome. One hypothesis is that, in some cultural contexts, intentionality is not considered to be a relevant element for the attribution of causality. According to this hypothesis, A → O is the most relevant link, with or without the I → O or I → A link, and whenever it is missing the relationship is seen as non-law-like. Another hypothesis is that, in contrast, mental states can be seen as adequate causes for physical events, so that, in the most extreme case, the I → O link is sufficient for the attribution of a causal relationship. This attribution of a causal relation between an intention and an outcome without obvious causal links involving physical actions can be seen as an example of “magical thinking.” Based on the fact that legal systems all over the world consider the actual actions of a person (and not his or her mental states) as important for convicting him or her, and based on psychological studies of causal attribution, we predict that in every culture the Action-to-Outcome (A → O) link will be the most important for attributing causation. However, the anthropological literature suggests that the principle of magical thinking might be more relevant in certain “non-western” cultural groups compared with those in “western” societies; i.e., although in most “western” cultures intentionality is important in blame attribution, it is less often considered a relevant causal factor. We therefore anticipate that intentionality might have more weight in the non-western samples. We will elaborate our predictions in Section Predictions and discuss this issue in relation to our results in Section Cross-cultural Comparison of the Conceptualization of Causality.

### The linguistics of causality

While the previous section was concerned with ideas and cultural preferences regarding concepts of causality, we want to emphasize the point that ideas and concepts are also (maybe sometimes even essentially) encoded in words. In every language, words and grammatical structures are not simply a tool for expressing pre-existing thoughts, but they also, to some extent, guide thinking processes (Sapir, 1933; Whorf, 1956; Lucy, 1992). For anthropologists as well as for linguists and cross-cultural psychologists, attention to lexical categories is crucial, for they represent “conceptual packages” with which speakers analyze and categorize their physical and social worlds. This point has been made extensively in the literature about color terms, for instance (Berlin and Kay, 1969; Hardin and Maffi, 1997; Levinson, 2000). Although colors can be objectively categorized using a color chart, color terms in any specific language cut the color space into categories, and different languages do this differently. The implications of this—including the extent to which the “linguistic relativity” hypothesis is valid (i.e., how people construe the world based on linguistic variations)—have been hotly debated (Lucy, 1992; Gumperz and Levinson, 1996).

Why is this debate important for a cross-cultural and cross-linguistic analysis of (exceptional) causality? The answer is simple: If people do not have a lexical label to express a concept like “chance,” “accident,” “coincidence,” they might not be able to interpret events that speakers of other languages construe as falling into those categories. Additionally, local folk theories might encourage the idea of a uni-causal interpretation. Thus, people from different cultural contexts and different language communities would give different explanations for the same situation. These explanations might or might not vary from each other in terms of causation but still be systematic within each community. How much people's judgments will be consistent and how much disparity there is across the interpretations of members of the same group were exactly the questions that drove our research.

## Materials and methods

Because our aim is to determine how participants from different cultures conceptualize causal links between events and, in particular, if they have some word or expression to relate events with each other in a non-law-like way, we designed a task that involved different scenarios under various conditions following a systematic structure. According to that structure eight scenarios represented different configurations of causal links, which were instantiated in eight cover stories providing different content to the causal structures (see details in Section Materials).

### Participants^4^

The German sample was composed of 64 participants, all students recruited from the campus of the University of Göttingen (Germany). Among the participants there were 32 women, 31 men, and one person who didn't specify his or her gender. The mean age was 24.97 (*SD* = 4.55). One person did not specify age. German participants were asked spontaneously on campus, mostly in cafeterias. They received sweets as compensation.

The Yucatec Maya sample was composed of 16 participants (nine women), all native speakers of Yucatec Maya; 14 were from the village of Kopchen (state of Quintana Roo) and two females from the village of Chican (state of Yucatán). Their mean age was 38.4 years (ranging from 18 to 50). None of the participants had more than a high school degree. Although all are native speakers of Yucatec Maya, some also speak Spanish. Yucatec Mayans participants were compensated indirectly through gifts, following a fieldwork procedure used since 2002 by Le Guen.

The Tseltal Maya sample was composed of 16 participants (eight women) all native speakers of Tseltal; all were from the community of Tenejapa (state of Chiapas). Their mean age was 32.8 years (ranging from 23 to 58). Education level varied; none had more than a high school degree. Tseltal participants volunteered in response to Brown's invitation to participate to the study. Each participant received 70 Mexican pesos per session (in which they also participated in other tasks).

The Mexican sample was composed of eight students from an undergraduate Spanish literature class at the UNACH University (Universidad Autonoma de Chiapas), studying for their Bachelor degree. Four of the students were female. All were from the state of Chiapas and all were native speakers of Spanish (one participant said he also speaks Mam (a Mayan language) but considers himself to be Mestizo, i.e., from the Mexican Spanish culture). The mean age of participants was 20.4 (between 18 and 24). Participants were offered candy or coffee as remuneration.

### Materials

We used the structural linkages of the *Culpable Control Model* by Alicke (2000) as the basis for designing our task. In this model, the relation between the intentionality of an agent, the action and the outcome is divided into three links: Intention-to-Outcome, Intention-to-Action, and Action-to-Outcome. The presence of the Intention-to-Outcome link (I → O) implies the desire of an agent that a certain event (the outcome) shall happen whereas its absence implies that the agent neither foresaw nor wanted the outcome to happen. The presence of the Intention-to-Action link (I → A) implies that the agent intended a certain action. This link can be present even though the Intention-to-Outcome link is absent and vice versa (see scenarios 3, 5, 6, and 7). Finally, the presence of the Action-to-Outcome link (A → O) implies that the action leads to a particular outcome. All combinations of the presence and absence of the mentioned three links lead to eight different scenarios, each with a unique pattern of links. Table 1 presents all eight combinations of the three possible links.

**Table 1:** **For each scenario (Sc), the structure considers the combination of the three possible links: Intention to Outcome (I → O), Intention to Action (I → A), and Action to Outcome (A → O)**.

	**English gloss**	**Links**		
		**I → O**	**I → A**	**A → O**
Sc1	“direct causality”	√	√	√
Sc2	“failure”	√	√	–
Sc3	“accident”	–	√	√
Sc4	“luck”	√	–	√
Sc5	“unintentional”	–	–	√
Sc6	“magical thinking”	√	–	–
Sc7	“intended action”	–	√	–
Sc8	“pure coincidence”	–	–	–

*The signs √ and – represent, respectively, the presence or the absence of a link*.

Scenarios 1 and 8 will be considered our baseline scenarios. Scenario 1, with all three causal links present, exemplifies a case of *direct causation*. For instance, consider the case of a successful event of killing a deer (our cover story 1). A hunter wants a dead deer (i.e., the I → O link is given, as there is an intention to an outcome), so he pulls the trigger with the purpose of shooting at the deer (i.e., the I → A link is given, as there is an intention to an action fulfilled). Eventually, the shot of the hunter leads to the dead deer (i.e., the A → O link is also given, as the action and the respective outcome are realized).

By contrast, scenario 8 is made up of purely *coincidental* events, that is, the three events just happen at the same time without any obvious causal link present between them, as, for example: A hunter goes into the forest and wants to clean his gun (i.e., no intention to kill the deer). While cleaning the trigger he accidentally pulls it. The gun doesn't fire because there was no bullet in the barrel. At the same moment a deer falls down, dead, some meters away from the hunter (i.e., no action from the agent leads to the outcome)^5^.

Scenario 2, where the link Action-to-Outcome is absent, is a typical case of *failure*, since intentionality is present but does not make the outcome happen. Scenario 3, which lacks the Intention-to-Outcome link, could be considered a case of *accident*, because there is an intention toward the action but no intention toward the outcome. Scenario 4, which lacks the Intention-to-Action link can be considered a prototypical case of *luck*. In the literature, this scenario has been referred to as a “deviant” causal chain (Chisholm, 1966; Searle, 1983; Pizarro et al., 2003). Scenario 5 can be referred to as *unintentional* as it represents a case in which neither the intention toward the action nor the intention toward the outcome is present, although, in the end, the outcome is caused by the action of the agent. Scenario 6, which presents only the Intention-to-Outcome link, is a case of *magical thinking*: the agent wants the outcome to happen and events (magically) turn out to comply with his or her wishes. Again, taking our cover story 1 as an example: A hunter wants a dead deer (i.e., link 1 is fulfilled, for there is an intention to an outcome). While walking in the forest he stumbles over a root and pulls the trigger accidentally. The gun does not fire because there was no bullet in the barrel (i.e., no intention leading to an action). At the same moment a deer falls down, dead, some meters away from the hunter (i.e., no action from the agent leads to the outcome). Finally, scenario 7 (*intended action*), with only the Intention-to-Action link, represents a situation in which an outcome happened that was neither intended nor was it the result of the agent's action.

What is important to note is that even while the eight scenarios differ regarding their constellation of linkages, there are three factors that remain constant across all scenarios: (a) there is always an agent mentioned whose behavior (event 1) is temporally and spatially near to the outcome (event 2), (b) the behavior of the agent leads to an event that happens constantly regardless of whether it is intended by the agent, and (c) all scenarios end with a similar outcome (e.g., the deer being dead, the window being broken, etc.).

Eight different cover stories were created, so that each scenario was combined once with each cover story leading to 64 different story-scenario-combinations. These were created to control for content effects; the stories have a different content but share the causal-link structure of the eight scenarios. With eight different scenarios and eight different cover stories we were able to vary possible combinations of the two to counterbalance our data in order to improve its reliability. The eight cover stories are the following:

1. A hunter shooting a deer
2. A boy kicking a ball and breaking a window
3. A fisher fishing a fish
4. A woman starting a fire
5. A woman breaking a plate, waking up her husband
6. A man spilling a drink on his boss
7. A man cutting down a cornstalk
8. A man killing an insect with a newspaper.

Stories were originally designed in English and then translated into the four languages. In the choice of contents, a main priority was to be as culturally neutral as possible. Alicke (Alicke, 2000; Alicke et al., 2008) was primarily interested in blame attribution, and in his model he recognizes the role of norms as fundamental as much as the valence of the outcome (positive or negative). This is why we avoided outcomes with strong valence (like human death or severe injuries), especially because human agents are involved in every story. Careful attention was taken to have culturally interpretable content of the stories also for the two Mayan groups^6^.

### Design and procedure

#### Design

The eight cover stories were designed in order to control for content effects. The strategy of assignment of the scenarios/cover stories to the participants differed across the four groups. For the Mexican Spanish, the Tseltal and the Yucatec participants, the full set of cover stories was used. Each participant got all eight scenarios, each with a different cover story, in a pre-randomized order that is presented in Appendix 1 of Supplementary Material. For the eight Mexican Spanish subjects this results in one data point for each scenario. For the two Mayan groups, after the first eight participants, the same structure was repeated with the next eight participants, resulting in two data points for each scenario. In Germany, only two stories (taken randomly from the eight cover stories) were presented to each participant since it was possible to recruit many more subjects compared to the other three groups. The two stories presented to a participant were randomly combined; the assignment was restricted in three ways: (1) every scenario for every cover story had to be assigned twice, one participant could neither (2) get two different scenarios for the same cover story nor, (3) the same scenario for two different cover stories. Each participant thus got two different scenarios with two different cover stories. The described procedure also results in two data points for each scenario.

#### Procedure

The scenarios were presented to the participants in their native tongue in a randomized order (given in Appendix 1 of Supplementary Material). In the case of the German students, the subjects were given the task on a sheet of paper and participants noted their answers down. For the other three groups, the cover stories/scenarios were presented orally; they were read as many times as necessary for the participant to understand them correctly. Participants answered verbally and their responses were noted on a note pad. Responses were also audio-recorded for the Mexican Spanish and the Tseltal and video recorded for the Yucatec.

Participants were asked to provide an interpretation of the (assumed) causal or other relation between the links for each scenario. They were asked three questions:

1. Temporal question: Why did the outcome occur just then?
2. Agency question: Did the actor cause the outcome to happen?
3. Counterfactual question: If the actor had not been there, would the outcome have happened anyway?

The first question was an open, temporal question on the timing of the outcome: “Why did the outcome occur just then?” The temporal criterion is fundamental in order to assess the coincidence of events. As pointed out by Hume (2003) and Lagnado and Channon (2008)^7^, people's attribution of causal relations can vary if events are considered earlier or later in the chain of events. As Alicke (2000) suggests, a closer proximity between action and the outcome might reveal a greater control by the agent and a higher degree of causality. This question, prompting for a free interpretation of the scenario, also enabled us to make a linguistic analysis of the concepts participants used to characterize the event described.

The second question focuses on the agency of the actor: “Did the actor cause the outcome to happen?”^8^. This was asked to determine whether participants recognize a causal relation between the intention or the action of the agent regarding the outcome.

The final question is formulated counterfactually: “If the actor had not been there, would the outcome have happened anyway?” This question was designed to determine how participants consider the agent to be determinant in the outcome (it contrasts directly with the agency question). It is important to point out that while the open question was aimed at eliciting explanations of the event, the two closed questions addressed directly the (causal) involvement of the agent in each scenario.

#### Coding

All answers were translated back into English in order to allow for multiple coders. Questions 2 and 3 triggered yes/no/I don't know answers and these three types of answers were considered. Question 1 was an open question, so answers were coded into one of six mutually exclusive categories according to the following criteria.

1. *Causal Story-based Explanations*. Answers in this category include a causal connection between the agent mentioned in the story (or a part of the mechanism between the agent's action and the outcome) and the outcome. When the Action-to-Outcome link was present (i.e., in scenarios 1, 3, 4, and 5), it suggests that participants recognized the causal connection between the agent's action and the outcome. By contrast, when the scenario structure did not have the Action-to-Outcome link, it suggests that the participant did not accept the scenario as such, but created a causal connection from the agent to the outcome although it was not originally present.
2. *Causal Imposed Explanations*. Answers in this category include an invented causal connection between a causal factor that was not mentioned originally in the story and the outcome. Examples of such answers are: “There was another hunter who shot the deer at the same time” (cover story 1) or “something hit the window, though it wasn't the ball” (cover story 2).
3. *No cause, it happened by itself, chance, coincidence*. Answers that belong to this category are those where the agent mentioned in the story has nothing to do with the outcome and no other causal mechanism is added by the participant in order to make sense of the story. Examples of such answers are: “it was chance that the deer died in that moment” (cover story 1) or “it fell down all by itself” (cover story 6).
4. *Fate, destiny*. Answers belonging to this category suggest that the outcome happened because it was “meant to be,” without the participant specifying any other causal mechanism. Examples of such answers are: “it was [the] destiny [of the deer to die]” or “it was [the fisher's] fate to catch [the fish], God took it out of the water so the fisher could catch it” (cover story 3). A typical word used in Yucatec Maya was *sweerte* “fate,” or equivalently, *Schicksal* “karma, fate, destiny” among the German participants.
5. *I don't know*. Answers belonging to this category suggest that the participant could not name a specific causal factor or could not categorize the story under a specific label. It is also the case that an “I don't know”-response reflects some degree of insecurity.
6. *Miscellaneous, not classifiable*. Answers that did not belong to any of the previous categories were coded as not classifiable. Such answers generally revealed that the participant did not answer the question or that the answer was unrelated to the question (e.g., “people will still say it's [the boy who broke the window]”) (cover story 2).

Because these were open answers, we decided to conduct a test of inter-rater reliability. The specialist of each cultural group coded the answers and translated them into English. A second coder blind-coded the first coder's answers, and, for cases in which the two raters did not agree, a third, independent rater decided which category the open answer in question was to be assigned to. For the German sample, the inter-rater reliability for the two raters was found to be excellent (κ = 0.97) according to Landis and Koch (1977). Reliability was lower, but nevertheless substantial agreement could be found both for the two raters of the Tseltal participants' answers (κ = 0.78) and for the two raters of the answers of the Yucatec subjects (κ = 0.68). For the Mexican Spanish participants, the inter-rater reliability for the two raters was only moderate (κ = 0.50). The differences in reliability partly reflect the extent to which a rater had prepared his or her coding task beforehand, but they also result of how much open answers were detailed. The answers of the German participants, for instance, were very detailed—perhaps because they were written down instead of orally given. It could therefore have been easier to classify them. However, the agreement between two raters on the assignment of categories was at least “substantial” for three of the four groups and the worst degree of agreement was still “moderate” (after Landis and Koch, 1977). We therefore consider the implementation of the coding system to be successful and that our use of the open answer-data is justified.

### Predictions

One main concern in this study is to explore the ways in which different cultural groups consider what we could consider “core or basic causality.” In particular, we are interested in the causal link between an Action and an Outcome (A → O), which is classically referred to as “causality” in Western societies. There are two possibilities: first, either all participants from every culture consider this link as fundamental or, alternatively, in some cultures this link is not taken to be so important in relation to other links (like Intention-to-Outcome or Intention-to-Action).

The Action-to-Outcome link determines whether an agent's action is seen as the cause of an outcome or not. The interest of considering the relevance of the Action-to-Outcome link for the interpretation of causality cross-culturally primarily lies in the fact that in the anthropological literature, it was a frequent claim among early ethnographers that members of many non-western cultural groups base a lot of their daily behavior on the principle of “magical thinking,” mostly related to various kinds of taboos (Frazer, 1911; Lévy-Bruhl, 1922; Evans-Pritchard, 1937; Lévi-Strauss, 1990; Malinowski, 1992), see discussion in section Cross-cultural Comparison of the Conceptualization of Causality. According to this notion, the other two links, (Intention-to-Outcome and Intention-to-Action) could likewise contribute to the perception of causality. If some cultural differences were to be expected, they would be between the German and the Mexican participants on the one hand, who should behave in the way expected of “western” groups, and the Tseltal and Yucatec participants on the other hand, who might show evidence of the kind of reliance on the I-O link typical of “magical thinking.”

## Results

We examine the results according to the three questions we asked our participants. For practical reasons, we consider first the agency question (Did the actor cause the outcome to happen?), then the counterfactual question (If the actor had not been there, would the outcome have happened anyway?) and finally the open, temporal question (Why did the outcome occur just then?). We look at both differences *within* cultures, depending on the absence or presence of each link (A → O, I → A, and I → O), and differences *between* cultures, given the presence of each link.

### The agency question

Answers to the question “Did the actor cause the outcome to happen?” reveal how much participants attribute causation to the actor in each scenario, and allow us to determine how much weight the different links are given in the recognition of causation. This question could be answered with “yes,” “no,” or “maybe.” A yes-answer would indicate that the agent is seen as cause of the outcome. For the calculation of the within-group contrasts, we used a 2 (link present vs. link absent) × 3 (response: yes/no/maybe) contingency table^9^. For the between-group contrasts, we used a 2 (group 1 vs. group 2) × 3 (yes/no/maybe) contingency table for the presence-case of each link (A → O, I → A, and I → O). The descriptive results are presented in Table 2.

**Table 2:** **Percentage of Yes-Answers to the question “Did the agent cause the outcome to happen?” for each language and for the presence and absence of each link**.

**Language**	**Percentage of Yes-Answers**					
	**A-O link present (sc. 1, 3, 4, and 5)**	**A-O link absent (sc. 2, 6, 7, and 8)**	**I-A link present (sc. 1, 2, 3, and 7)**	**I-A link absent (sc. 4, 5, 6, and 8)**	**I-O link present (sc. 1, 2, 4, and 6)**	**I-O link Absent (sc. 3, 5, 7, and 8)**
German	79.69	21.88	40.63	60.94^a^	54.69	46.88
Tseltal	57.81	29.69	50.00	37.50	54.69	32.81
Yucatec	89.06	42.19	73.44	57.81	67.19	64.06
Mexican Spanish	59.38	25.00	37.50	46.88	46.88	37.50

*Note that each scenario was answered by 16 German, 16 Tseltal, 16 Yucatec, and 8 Mexican Spanish participants so that the percentages in each column refer to 64 German, 64 Tseltal, 64 Yucatec, and 32 Mexican Spanish participants*.

a *The German and Mexican Spanish subjects gave more “yes” answers in the absence compared to the presence of the I → A link. This difference can be explained by the presence or absence of the A → O link: there generally tend to be more “yes” answers for those scenarios in which the A → O link is present (1, 3, 4, 5) and more “no” answers in those in which the A → O link is absent (2, 6, 7, 8). Regarding the four scenarios in which the I → A link is absent, for instance, the higher percentage of “yes” answers can solely be attributed to the two scenarios 4 and 5 in which the A → O link is present (German subjects: 15 “yes” and 1 “no” answer to scenario 4, 13 “yes” and 2 “no” answers to scenario 5, 6 “yes” and 6 “no” answers to scenario 6, 5 “yes” and 10 “no” answers to scenario 8; Mexican Spanish subjects: 5 “yes” and 3 “no” answers to scenario 4, 5 “yes” and 3 “no” answers to scenario 5, 2 “yes” and 6 “no” answers to scenario 6, 3 “yes” and 4 “no” answers to scenario 8)*.

#### Comparison within cultures

For subjects of all four cultural backgrounds, the only significant differences between the absence and the presence of a link were found for the A → O link: if it is present, the agent is significantly more often seen as cause compared to when it is absent [German: $\chi_{\left(2,\;N=128\right)}^{2}$^10^ = 43.51; *p* < 0.001, Tseltal: $\chi_{\left(2,\;N=128\right)}^{2}$ = 10.86; *p* = 0.004, Yucatec: $\chi_{\left(2,\;N=128\right)}^{2}$ = 31.38; *p* < 0.001, Mexican Spanish: $\chi_{\left(2,\;N=64\right)}^{2}$ = 9.36; *p* = 0.009]. Only for the Tseltal subjects, a second link seems to have been important in order to answer the question: the I → O link. They stated significantly more often that the agent did not cause the outcome if the outcome was not intended compared to when it was intended [$\chi_{\left(2,\;N=128\right)}^{2}$ = 6.88; *p* = 0.03].

So as predicted, for the participants of all four cultural backgrounds the most important link to decide whether an agent caused the outcome is the link from the agent's action to the outcome. However, there could be differences regarding the importance of the links between the participants of the different cultural backgrounds; that the agent's action caused the outcome, for instance, could still be more important for some than for others.

#### Comparison between cultures

To see whether there are differences in the relative importance of the three links between participants of the four cultural backgrounds, we analyzed the differences between every pair of groups, resulting in six comparisons: German–Tseltal, German–Yucatec, German–Mexican Spanish, Tseltal–Yucatec, Tseltal–Mexican Spanish and Yucatec–Mexican Spanish^11^.

If the A → O link is present, the vast majority of the German subjects see the agent as cause (79.69%). Their answer pattern is different from that of the Tseltal and Mexican subjects [German–Tseltal: $\chi_{\left(2,\;N\;=\;128\right)}^{2}$ = 17.54; *p* < 0.001, German–Mexican Spanish: $\chi_{\left(2,\;N\;=\;96\right)}^{2}$ = 11.42; *p* = 0.003]. A consideration of the adjusted standardized residuals^12^ revealed that these differences were due to the preponderant majority of German subjects endorsing the agent as a cause compared to more evenly distributed answers in the Tseltal and Mexican-Spanish samples and, at least for the German-Tseltal comparison, due to more “maybe”-answers on the part of the German subjects. The answer pattern of the Yucatec subjects resembles that of the Germans (the general answer pattern did not differ significantly; $\chi_{\left(2,\;N=128\right)}^{2}$ = 5.4; *p* = 0.067); the adjusted standardized residuals merely revealed that the Germans gave more maybe-answers compared to the Yucatec sample (see Appendix 2 in Supplementary Material). Also the Yucatec–Tseltal and the Yucatec–Mexican comparison revealed significant differences: Yucatec subjects less often deny and more often state that the agent is the cause if the A → O link was present in comparison with the Tseltal subjects [$\chi_{\left(1,N=128\right)}^{2}$ = 16.02; *p* < 0.001] or the Mexican Spanish subjects [$\chi_{\left(1,\;N=96\right)}^{2}$ = 11.4; *p* < 0.001].

If the I → A link is present, the answer pattern of the German subjects differs significantly from that of the Tseltal subjects [$\chi_{\left(2,\;N=128\right)}^{2}$ = 12.04; *p* = 0.002]. The adjusted standardized residuals indicate that this difference stems from more “maybe”-answers of the German subjects. This finding, however, might be due to differences in how the data were collected: the German subjects were given a written questionnaire with “maybe” as an answer option whereas the Tseltal subjects were asked to answer verbally and thus the answer “maybe” might not have come readily to their mind. For the Yucatec participants, the agent is more often seen as the cause of the outcome if he intended the action than for the German and Mexican subjects (German–Yucatec: $\chi_{\left(2,\;N=128\right)}^{2}$ = 17.19; *p* < 0.001, Yucatec–Mexican $\chi_{\left(2,\;N=96\right)}^{2}$ = 11.87; *p* = 0.003). The German participants, additionally, gave more “maybe”-answers compared to the Yucatec participants, as the adjusted standardized residuals indicate.

Regarding the I → O link, only the German subjects seem to have given a slightly different answer pattern compared to the Tseltal [$\chi_{\left(2,\;N=128\right)}^{2}$ = 13.57; *p* = 0.001]. This is, as the analysis of the adjusted standardized residuals indicates, again due to the higher frequency of maybe-answers from the German subjects.

#### Summary

The results from the agency question overall show that intentionality does not play the major role for attributing causality to an agent, at least among these four cultural groups, while the A → O link seems to be the most important one for determining whether an agent is the cause of an outcome. However, there are differences between participants from the four cultural backgrounds: compared to the German and Yucatec subjects, the Tseltal and Mexican Spanish subjects deny the agent's causal role more often even when the story is more likely to represent the agent's action as causing the outcome. In addition, compared to the other three groups the Yucatec participants see the agent more often as cause even if he merely intended the action. For some cultural groups, the intentionality of an action therefore seems to play an additional role in their causal attributions.

### Counterfactual factor

The counterfactual question (“If the actor had not been there, would the outcome have happened anyway?”) was designed to test whether counterfactual evidence would cancel a causal interpretation. Possible answers for this question were again “yes,” “no” or “maybe.” Note, however, that the representation of the agent as cause of the outcome would be indicated by a negation of the question (“No, the outcome would not have happened without the agent being there”). The within-contrasts were again calculated using a 2 (link present vs. link absent) × 3 (response: yes/no/maybe) contingency table (Tseltal and Yucatec participants were less likely to answer “maybe”; see footnote 8). The between-contrasts, again, were calculated using a 2 (group 1 vs. group 2) × 3 (yes/no/maybe) contingency table for the presence-case of each link (A → O, I → A, and I → O). The descriptive results are presented in Table 3.

**Table 3:** **Percentage of No-Answers to the question “If the actor had not been there, would the outcome have happened anyway?” for each language and for the presence and absence of each link**.

**Language**	**Percentage of No-Answers**					
	**A–O link present (sc. 1, 3, 4, and 5)**	**A–O link absent (sc. 2, 6, 7, and 8)**	**I–A link present (sc. 1, 2, 3, and 7)**	**I–A link absent (sc. 4, 5, 6, and 8)**	**I–O link present (sc. 1, 2, 4, and 6)**	**I–O link absent (sc. 3, 5, 7, and 8)**
German	75.00	25.00	43.75	56.25^a^	50.00	50.00
Tseltal	100.00	78.13	89.06	89.06	89.06	89.06
Yucatec	84.38	39.06	65.63	57.81	56.25	67.19
Mexican Spanish	68.75	40.63	43.75	65.63	62.50	46.88

*Note that each scenario was answered by 16 German, 16 Tseltal, 16 Yucatec, and 8 Mexican Spanish participants so that the percentages in each column refer to 64 German, 64 Tseltal, 64 Yucatec and 32 Mexican Spanish participants*.

a As already noted for question 2, the German and Mexican Spanish subjects gave more “yes” answers to question 3 if the I → A link was absent compared to when it was present. The Yucatec subjects gave more “yes” answers if the I → O link was absent compared to when it was present. These differences can predominantly likewise be also be attributed to the presence of the A → O link. This link was present in two of the four scenarios in which the I → A link was absent, scenarios 4 and 5, and also in two of the four scenarios in which the I → O link was absent, scenarios 3 and 5. (German subjects: 1 “yes” and 13 “no” answers to scenario 4, 3 “yes” and 13 “no” answers to scenario 5, 7 “yes” and 3 “no” answers to scenario 6, 7 “yes” and 7 “no” answers to scenario 8; Mexican Spanish subjects: 0 “yes” and 7 “no” answers to scenario 4, 1 “yes” and 5 “no” answers to scenario 5, 0 “yes” and 6 “no” answers to scenario 6, 2 “yes” and 3 “no” answers to scenario 8; Yucatec subjects: 2 “yes” and 14 “no” answers to scenario 3, 2 “yes”—and 14 “no” answers to scenario 5, 8 “yes” and 8 “no” answers to scenario 7, 9 “yes” and 7 “no” answers to scenario 8.)

#### Comparison within cultures

As for the agency question, the only significant differences between the absence and presence of one link can be found for the A → O link. If the agent's action caused the outcome, more participants say that the outcome would *not* have happened without the agent's presence than that it *would* have happened without him. This difference is significant for the German subjects [$\chi_{\left(2,\;N=128\right)}^{2}$ = 33.91; *p* < 0.001], for the Tseltal subjects [$\chi_{\left(2,\;N=128\right)}^{2}$ = 15.72; *p* < 0.001], for the Yucatec subjects [$\chi_{\left(1,\;N=128\right)}^{2}$ = 27.81; *p* < 0.001] and marginally significant for the Mexican Spanish subjects [$\chi_{\left(2,\;N=64\right)}^{2}$ = 5.48; *p* = 0.06].

The responses of the majority of subjects of all cultural backgrounds indicate that, in cases in which the A → O link is present, the outcome would not have happened if the agent had not been there.

#### Comparison between cultures

For both the Tseltal and the Yucatec subjects, the comparisons with the other cultural groups revealed significant differences if the A → O link is present. All Tseltal participants denied that the outcome would have happened without the agent and thus gave more no-answers and less maybe-answers than the German subjects [$\chi_{\left(2,\;N=128\right)}^{2}$ = 18.29; *p* < 0.001], although the majority of German subjects also answered “no” (75%). For the same reason (because of the large amount of no-answers on the part of the Tseltal), the comparison with the Yucatec and Mexican subjects also reveals significant differences [Tseltal–Yucatec: $\chi_{\left(1,\;N=128\right)}^{2}$ = 10.85; *p* < 0.001, Tseltal-Mexican: $\chi_{\left(2,\;N=96\right)}^{2}$ = 22.34; *p* < 0.001]. In addition, the Yucatec subjects' answer pattern differs significantly from that of the Germans [$\chi_{\left(2,\;N=128\right)}^{2}$ = 11.35; *p* = 0.003] and Mexican subjects [$\chi_{\left(2,\;N=96\right)}^{2}$ = 15.27; *p* < 0.001]: both the German and the Mexican Spanish participants gave more maybe-answers than the Yucatec participants, as the analysis of the adjusted standardized residuals revealed.

So, given the presence of the A → O link, *all* Tseltal subjects answered “no” to the counterfactual question as to whether the outcome would have happened if the agent had not been there. The Yucatec participants sometimes answered “yes,” and only the German and Mexican participants also answered “maybe” (although rarely).

Regarding the importance of the I → A link, again for both the Tseltal and the Yucatec subjects, the comparisons with the other cultural groups revealed significant differences concerning their answers if the I → A link is present. Compared to the German subjects [$\chi_{\left(2,\;N=128\right)}^{2}$ = 29.48; *p* < 0.001], the Yucatec subjects [$\chi_{\left(2,\;N=128\right)}^{2}$ = 20.71; *p* < 0.001] and the Mexican subjects [$\chi_{\left(2,\;N=96\right)}^{2}$ = 22.75; *p* < 0.001], the Tseltal subjects gave significantly more no-answers if the agent intended his action, suggesting that he was seen to be a *causal* agent based on the presence of the I → A link. As likewise indicated by the adjusted standardized residuals, the German and Mexican Spanish participants also gave more maybe-answers compared to the Tseltal participants.

Also for the Yucatec subjects, however, the presence of the I → A link seems to influence the representation of the agent as cause in a stronger way than for the German [$\chi_{\left(2,\;N=128\right)}^{2}$ = 22.44; *p* < 0.001] and Mexican subjects [$\chi_{\left(2,\;N=96\right)}^{2}$ = 22.35; *p* < 0.001]. Compared to them, the adjusted standardized residuals show that the Yucatec participants gave more no-answers and fewer maybe-answers—indicating that they considered the agent to be “more causal” if the I → A link was present.

Finally, as for the other two links, the comparisons between the Tseltal and the Yucatec subjects with all other cultural groups revealed significant differences concerning their answers if the I → O link is present. The Tseltal subjects denied significantly more often that the outcome would have occurred without the agent if the outcome was intended by the agent compared to the German [$\chi_{\left(2,\;N=128\right)}^{2}$ = 23.19; *p* < 0.001], Yucatec [$\chi_{\left(2,\;N=128\right)}^{2}$ = 25.74; *p* < 0.001] and Mexican participants [$\chi_{\left(2,\;N=96\right)}^{2}$ = 9.93; *p* = 0.007]. The role of the link between intention and outcome therefore seems to be most important for the Tseltal subjects: if the I → O link is present, the agent is seen as “more causal.” The German and Mexican subjects, again, also gave more maybe-answers than the Tseltal participants. Interestingly, the Yucatec subjects gave more yes-answers and fewer maybe-answers than the Mexican subjects [$\chi_{\left(2,\;N=96\right)}^{2}$ = 19.05; *p* < 0.001] and the German subjects [$\chi_{\left(2,\;N=128\right)}^{2}$ = 25.74; *p* < 0.001] as the adjusted standardized residuals reveal. This indicates that, compared to the German and Mexican subjects, the agent is “less causal” for the Yucatec participants if the I → O link is present. However, the Yucatec participants did not give fewer no-answers compared to these two samples (see Appendix 2 in Supplementary Material)—which would be the necessary counterpart for this conclusion—suggesting that this result might be an artifact resulting from the general tendency of the Yucatec participants to not give maybe-answers.

#### Summary

For the participants of all cultural backgrounds, the A → O link was the most important link to determine whether the outcome would have happened in the absence of the agent. However, there were differences across the four groups. Whereas for the German and Mexican subjects, the presence of the A → O link seems to have been the only relevant information for answering the counterfactual question, the Yucatec participants and even more so the Tseltal participants seem to have considered the other two links as well for their judgment. This can be interpreted as an influence of the story agent's mental state on the participant's causal representation of the event. Also the finding of the agency question supports this interpretation: even if the agent's action caused the outcome, Tseltal and Yucatec participants seem to be more willing to say that the agent is not the cause of the outcome. This could be because, for them, the agent's intentionality toward the action and the outcome plays a bigger role than for the German and Mexican participants.

However, there is a pattern in the Tseltal data—a strong contrast between the responses to the agency question and the counterfactual question—that differs from that for all three of the other cultures. The Tseltal responses to the agency question more rarely attributed causality to the agent compared to German and Yucatec responses (i.e., they provided more no-answers), suggesting that the agent is not seen to be as much a source of causality as in the data of the German or Yucatec participants. Yet the majority of Tseltal responses to the counterfactual question support the idea across all scenarios that the event could only have happened if the agent were present. In other words, they appear to be seeing the agent as less responsible in the first case but as a prerequisite for the outcome to happen in the second case. This unique pattern for Tseltal suggests the possibility that Tseltal participants took a different perspective in the counterfactual case, for example they might have viewed the agent as an essential witness of the scenario who is important for the story to be perceived and retold, and therefore, the agent might be a prerequisite for each scenario^13^. What exactly the implications are of this Tseltal response pattern for Tseltal understandings of causality and agency clearly requires further research.

### The temporal question

The temporal question “Why did the outcome occur just then?” aimed at generating an open answer. As mentioned, the time criterion was crucial to avoid participants inferring other potential causal links that were not provided in the original story. The open answers participants gave were categorized in one of six categories: (1) causal-story based, (2) causal-imposed, (3) chance, (4) fate, (5) I don't know, and (6) miscellaneous. A causal representation of the agent would clearly be indicated by the first category (see Section Materials for details). For the calculation of the within-contrasts, we used a 2 (link present vs. link absent) × 6 (type of explanation: causal-story based, causal imposed, fate, chance, don't know, miscellaneous) contingency table with 12 cells for each language group. For the between-contrasts, we used a 2 (group 1 vs. group 2) × 6 (type of explanation: causal-story based, causal imposed, fate, chance, don't know, miscellaneous) contingency table for the presence-case of each link. The results are presented in Table 4.

**Table 4:** **Percentage of responses to the question “Why did the outcome occur just then (i.e., at that very moment)?” according to major categories of responses for each language and for the presence and absence of each link**.

**Language**	**A–O-link**	**Type of response in %**					
		**Causal-story based**	**Causal-imposed**	**Chance**	**Fate**	**I don't know**	**Miscellaneous**
German	Present	75.00	0.00	12.50	1.56	1.56	9.38
	Absent	18.75	28.13	20.31	9.38	12.50	10.94
Tseltal	Present	92.19	1.56	1.56	0.00	3.13	1.56
	Absent	62.50	17.19	14.06	0.00	4.69	1.56
Yucatec	Present	67.19	7.81	3.13	12.50	1.56	7.81
	Absent	15.63	42.19	1.56	28.13	6.25	6.25
Mexican Spanish	Present	75.00	12.50	6.25	0.00	0.00	6.25
	Absent	34.38	43.75	12.50	0.00	0.00	9.38
**Language**	**I–A-link**	**Type of response in %**					
		**Causal-story based**	**Causal-imposed**	**Chance**	**Fate**	**I don't know**	**Miscellaneous**
German	Present	43.75	18.75	15.63	4.69	4.69	12.50
	Absent	50.00	9.38	17.19	6.25	9.38	7.81
Tseltal	Present	75.00	9.38	9.38	0.00	3.13	3.13
	Absent	79.69	9.38	6.25	0.00	4.69	0.00
Yucatec	Present	45.31	23.44	1.56	15.63	4.69	9.38
	Absent	37.50	26.56	3.13	25.00	3.13	4.69
Mexican Spanish	Present	46.88	37.50	6.25	0.00	0.00	9.38
	Absent	62.50	18.75	12.50	0.00	0.00	6.25
**Language**	**I–O-link**	**Type of response in %**					
		**Causal-story based**	**Causal-imposed**	**Chance**	**Fate**	**I don't know**	**Miscellaneous**
German	Present	48.44	10.94	14.06	6.25	6.25	14.06
	Absent	45.31	17.19	18.75	4.69	7.81	6.25
Tseltal	Present	73.44	14.06	6.25	0.00	4.69	1.56
	Absent	81.25	4.69	9.38	0.00	3.13	1.56
Yucatec	Present	46.88	23.44	0.00	20.31	1.56	7.81
	Absent	35.94	26.56	4.69	20.31	6.25	6.25
Mexican Spanish	Present	50.00	28.13	9.38	0.00	0.00	12.50
	Absent	59.38	28.13	9.38	0.00	0.00	3.13

Note that each scenario was answered by 16 German, 16 Tseltal, 16 Yucatec, and 8 Mexican Spanish participants so that the percentages in each column refer to 64 German, 64 Tseltal, 64 Yucatec, and 32 Mexican Spanish answers

#### Comparison within cultures

As in the responses to the other two questions, the A → O link seems to be the most crucial one for the participants of all cultural backgrounds when it comes to their causal representation of the scenario. The answer pattern of all groups differed significantly when scenarios in which the agent's action caused the outcome are compared with those in which it does not [German: $\chi_{\left(5,\;N=128\right)}^{2}$ = 49.88; *p* < 0.001, Tseltal: $\chi_{\left(4,\;N=128\right)}^{2}$ = 18.58; *p* < 0.001, Yucatec: $\chi_{\left(5,\;N=128\right)}^{2}$ = 41.76; *p* < 0.001, Mexican Spanish: $\chi_{\left(2,\;N=64\right)}^{2}$ = 11.25; *p* = 0.01]. This is most likely because of more answers categorized as “causal-story based” in the first compared to the latter case.

#### Comparison between cultures

If the A → O link is present, the answer pattern of the Tseltal subjects differs significantly from that of the Yucatec subjects [$\chi_{\left(5,\;N=128\right)}^{2}$ = 16.51; *p* = 0.005]. The analysis of the adjusted standardized residuals shows that the Tseltal subjects more often give a causal-story based answer compared to the Yucatec subjects, whereas the Yucatec subjects give more fate-answers. The comparison between all other groups revealed no significant differences (all χ^2^ < 14.42, all *p*>0.013, i.e., higher than the necessary *p*-value of 0.008; see footnote 10).

If we now consider the I → A link, we notice that again, the answer pattern of the Tseltal subjects differs significantly from that of the Yucatec subjects [$\chi_{\left(5,\;N=128\right)}^{2}$ = 24.32; *p* < 0.001], and also from that of the Mexican Spanish subjects [$\chi_{\left(4,\;N=96\right)}^{2}$ = 14.42; *p* = 0.006]. According to the adjusted standardized residuals, this difference can likely be attributed to the higher amount of causal-story based answers and the lower amount of causal-imposed answers on the part of the Tseltal subjects compared to the other two groups. Moreover, the Yucatec participants gave more fate-answers than the Tseltal participants (who never gave a fate answer, actually).

Finally, for the I → O link, as for the presence of the other two links, the Tseltal participants' answer pattern differs significantly from those of the Yucatec subjects [$\chi_{\left(5,\;N=128\right)}^{2}$ = 25.92; *p* < 0.001] and the German subjects [$\chi_{\left(5,\;N=128\right)}^{2}=16.0$; *p* = 0.007]. Again, looking at the adjusted standardized residuals suggests that this is because of more causal-story based answers by the Tseltal subjects. For the comparison between the Tseltal and the Yucatec subjects, the significant difference additionally stems from the higher number of fate-answers on the part of the Yucatec participants. In addition, the German subjects gave more miscellaneous-answers compared to the Tseltal subjects. The answer pattern of the German and Yucatec participants also differs significantly [$\chi_{\left(5,\;N=128\right)}^{2}$ = 19.63; *p* = 0.001]. The adjusted standardized residuals reveal that the two groups do not differ regarding the amount of given causal-story based answers, but rather regarding some other answers: whereas the German participants give some chance answers, the Yucatec subjects more often give causal-imposed and fate answers.

#### Summary

The clearest finding regarding the open answers the participants gave in response to the temporal question is that the A → O link determines whether a causal-story based answer is given or not. It is interesting, however, that the Tseltal subjects give many causal-story based answers irrespective of this link—depending on the mental state of the agent (intention toward the action and intention toward the outcome). These findings seem to reflect in part the findings for the agency and the counterfactual questions, for which it can likewise be concluded that, for the Tseltal participants, mental states play a bigger role in the identification of causality. For the Yucatec participants, this conclusion can probably be drawn from the results of the agency and counterfactual questions but not for the open temporal question. Regarding the Yucatec, it is interesting that fate seems to be an adequate explanation in several cases, whereas neither the Tseltal nor the Mexican Spanish participants gave fate answers.

## General discussion

In this section we first present a summary of the main results of our study with regard to the predictions made in Section Predictions. Then, we point out some limitations of our study. We also propose a linguistic analysis of the answers from the open question before entering into a discussion of the cross-cultural comparison of the conceptualization of causality, looking in particular at the issue of the “magical thinking” principle from a cross-cultural perspective.

### Summary of the results and answers to predictions

In Section Predictions we presented a set of predictions which we can now compare to our cross-cultural results. Regarding the first prediction about the importance of the Action-to-Outcome-link, the reported findings suggest that, for the participants of all four groups, this link is indeed the most crucial one for the attribution of causality. Within each group and for all three questions, this link determines whether the agent is seen as cause (agency question), whether the outcome would have happened even without the agent (counterfactual question) or whether a causal story based answer is given or not (open, temporal question). It can be concluded therefore that in general, people from very different cultural backgrounds base their causal attributions on more or less the same “mechanistic” principle, i.e., whether there was a causal mechanism (an action leading to an outcome in our examples) that produced an outcome.

However, there are also differences between the answers of the four cultural samples we compared that might shed light on the validity of our second prediction, that is, if in every culture the Action to Outcome link is equally important for recognizing causation. It seems that the role of intentionality for the perception of causality differs slightly across the four groups. Whereas the German participants seem to attribute causality to the agent whenever the A → O link is present, the Tseltal and Yucatec participants sometimes do not see the agent as cause although his action led to the outcome—depending on whether intentionality to the action or to the outcome was present or not. In particular, the open answers of the Tseltal subjects reveal that causal story based answers were not limited to the presence of the A → O link.

To sum up, these findings suggest that for the attribution of causality, mental states like intentionality play a bigger role for the Tseltal and Yucatec participants compared with the German and Mexican Spanish subjects. Interestingly however, the intentional dimension is not present in the linguistic answers of the participants, as detailed in Section Linguistic Analysis of Open Answers below.

### Limitations of the study

Because our study is original and exploratory in various aspects, it has some limitations that we would like to point out for further comparative work.

First, we designed eight scenarios with abstract structures that were filled with different cover stories in order to prevent the task from being confusing or annoying for participants. Despite our best efforts, it seems that content did influence to some extent the interpretation of certain scenarios. However, this limitation is not critical for our study for two reasons. First, we could detect some content effects, as in the case of dreams for the Yucatec Mayas: some participants said, for example, that the man woke up because he dreamt of the event about to happen, which is, in accordance with the local concept of “fate”^14^. Second, content effects were minimized because each abstract structure was filled with different cover stories and tested with various participants (see Appendix 1 of Supplementary Material on the structure of the stories).

Sample size is another limitation that was almost inevitable in our case. It should be noted that recruiting willing participants in non-western nonacademic contexts is difficult and time-consuming. Our results, however, can be seen as primary data and future work can build on these findings.

Finally, a factor that could have been a limitation is that, while for both Maya populations and for the Mexican one the answer categories to the agency and counterfactual questions were read to them only once at the beginning of the task, for the Germans it was printed and thus available. It is possible therefore that “maybe” was not as salient as a possible answer as in the printed version. However, other studies run among the same Mayan groups by the same researchers would seem to point to the fact that not using “maybe” as an answer is habitual (Le Guen, 2006; Le Guen and Pool Balam, 2012).

### Linguistic analysis of open answers

The question that drove this study is whether people in different cultural groups have a similar understanding of causality and whether and how different cultural groups conceptualize exceptional, non-law-like relations between events (see Ojalehto and Medin, 2015 for a review). Specifically, we wanted to establish whether people from cultural settings other than the familiar Indo-European ones have concepts like “chance,” “coincidence,” or any other way to characterize non-obvious causal relations. The results from our comparative study in four cultures through the open, temporal question show that the construal of causality is culturally and linguistically driven. We found that German and Mexican students express non-law-like relations between events using concepts such as *Zufall* or *casualidad* (“(by) chance”), but neither of the Mayan groups expressed this idea, instead expressing the same events in a different way. Further, although the Mayan groups are culturally and linguistically related, they seem to have different ideas when judging non-law-like relations between events. Although both Mayan groups seem to put more emphasis on agency, Tseltal Mayas tend to segment a causal link into micro-causal links, i.e., enabling conditions that are distinct from the mind of the agent; they use the concept of *y-oloj* “by itself,” “of its own volition” to suggest, for example, that the machete chopped the cornstalk down of its own accord without any input from the man. Yucatec Mayas, in contrast, tend to regard all events as predetermined and ultimately dictated by fate *(sweerte)* and God's will, i.e., independently of the mind of an agent (or guiding it without his or her knowledge). We turn now to the main concepts used in open answers in each language.

#### The german notion of *Zufall*

In German, the notion of *Zufall* covers various concepts glossed in English as “chance,” “randomness,” “accident” but also “coincidence.” In the responses of German participants, *Zufall* was used with all of those meanings.

In the German answers to the open, temporal question, the concept of *Zufall* was used when some links between events were missing, in particular if the Action to Outcome was realized without any intentionality. However, *Zufall* was also used if there was only Intentionality to Outcome but without an Action to Outcome link. Participants seem to have used it as an explanation in non-causal scenarios to imply that there was “no (obvious) cause” to the outcome (e.g., scenario 4), similarly to the English idea of “coincidence,” but also in causal scenarios to imply that the outcome was not intentional (like in scenario 6), closer to the notion of “accident” in English. Sometimes *Zufall* was used to express the realization of the cause itself. For instance, one answer to the question of why the outcome happened when the action took place in Scenario 7 was literally “through, by, or due to *Zufall*.” Another answer to Scenario 2 was: “[it happened] because of *Zufall*.” Such answers convey the heterogeneous meanings of *Zufall* in German to express the recognition of exceptional causation: on the one hand it is used to express the absence of a cause in a given scenario, on the other hand it is used—linguistically—to express a kind of cause.

#### Notions of coincidence and chance in spanish

Spanish, like other Indo-European languages, has several ways of expressing the notion of non-law-like causal relations. Mexican participants used the words *coincidencia* “coincidence” (sc. 2, 3), *casualidad* “(by) chance” (sc. 4, 6), *buena suerte* “good luck” (sc. 7, 8) or *accidentalmente* “accidentally, by accident” (sc. 5, 7). In this respect Spanish is not significantly different from English or German. Because the language has words to express cultural concepts of non-law-like relations between events, participants have the resources to classify these events in comparable categories.

#### Yucatec maya and the notion of sweerte “fate”

There is no native lexicon in Yucatec Maya that relates to a notion of non-law-like relations between events like “chance” or “coincidence.” Lexical categories of this kind are borrowed from Spanish, and have been semantically altered in the process from their meanings in the source language.

One crucial notion is the one of *sweerte* “chance-fate.” The word *sweerte* in Maya comes from the Spanish *suerte* meaning “luck,” “chance” or “fortune.” However, when borrowed into Maya, the term refers to some kind of chance but more generally implies “fate.” Although *sweerte* in Maya can mean chance, it seems that ultimately, Yucatec Mayas consider that everything is meant to be, i.e., predetermined, so “good, bad or dumb luck” is written or determined by God. It is not uncommon to hear in everyday conversation regarding positive but also dramatic events (e.g., someone marrying an old lover or someone falling from a ladder to end up dead) the following expression: *bey usweerte máak* “that's people's fate” or *usweerte beya* “it was his/her fate like this,” meaning that what happened to the person in question was his/her fate regardless of circumstances or his/her will. This idea was very explicit in many Yucatec Mayan participants' responses as well as in interviews conducted after the task: although the first meaning “luck” actually refers to “punctual luck” (e.g., the hunter while cleaning his gun, shoots the deer), ultimately, more detailed explanation leads participants to say that it was fate. So luck is only a superficial reading of the event and not an explanatory recourse, ultimately everything can be explained by fate. In the counterfactual case, some participants agreed that the deer would have died anyway, maybe not in these particular circumstances but it would have died at this particular moment and the hunter would have killed a deer, maybe not this particular deer, but one deer.

In Yucatec Maya there is no word that encodes the concept of coincidence, although there are ways to express non-law-like relations (e.g., pointing to the simultaneity of events). One way is to use terms like “to think” or “to guess” with negation to refer to unplanned or fortuitous events. For Yucatec Mayas, foreseeing events or places (i.e., precognition) is considered to be actually possible. It is common to listen to people talking about dreams they have had about future events or distant places (see Groark, 2009 for a similar analysis among the Tsotsil Mayas).

#### The tseltal language of causality and non-causal events

Tseltal has a range of ways of expressing “no causal outcome.” Although there are no words in Tseltal for “by chance” or “accidentally,” related ideas can be expressed using other expressions such as *jowil* “for no reason, to no (good) purpose,” *ma'yuk y-ajwal* “there was no “owner” (of the deed), no one made it happen,” or *s-tukel* “by itself, without external agent.”

In contrast to Yucatec Maya, however, Tseltal Mayas in this task did not express strong views about fate or predetermined outcomes as an explanation for events. Instead, answers from Tseltal participants tended to decompose causal links into smaller causal chains. In particular, they used constructions with *y-oloj* which can be translated as “on purpose, deliberately, of his/its own volition.” While prototypically this term is used to explicitly attribute intentionality to an agent (“He did it on purpose”), interestingly—and this is where the semantics differs from the English glosses—even inanimate things can make things happen “on purpose” or “by their own volition.” The expression *y-oloj* is somewhat close to English “responsibility”—who is to be held responsible for making the thing happen. This expression is usually used to attribute responsibility for something bad happening, and differs from English “responsibility” in that it can apply to inanimates. For instance, one's heart will be “responsible” if one has a heart attack or it will be the mud, if one falls in the mud, etc. (see also Polian, forthcoming).

Tseltal participants had no difficulty in not attributing intentionality to the actor described in the task scenarios; they tended to generally break causal links into smaller ones suggesting that the presence of the agent's intention is not necessary to their interpretation. Hence in scenarios where the Action to Outcome link is not present (scenarios 2, 6, 7, and 8), Tseltal participants tended to use *y-oloj* “on its own responsibility,” bypassing the agent in favor of another element in the event chain to characterize non-intentional causality. Using this concept of *y-oloj* in these contexts seems to skip over the mental state (they do not need to pay attention to the agents' intentions) and attribute causal force to another link in the chain (e.g., to the instrument, or to the conditions in which the event occurred).

### Cross-cultural comparison of the conceptualization of causality

As already mentioned, it was frequently claimed by early ethnographers that members of many non-western cultural groups base a lot of their daily behavior on the principle of “magical thinking,” mostly related to various kinds of taboos (Frazer, 1911; Lévy-Bruhl, 1922; Evans-Pritchard, 1937; Lévi-Strauss, 1990; Malinowski, 1992). It was especially emphasized by Evans-Pritchard that not only do assumed causes for specific events differ across cultures, but also the coincidence of selected events (for example, people sitting down, a granary collapsing) require an explanation. For people in most “western” cultures, it might be seen as “bad luck” for the particular Azande individuals who happened to be sitting beneath a granary which suddenly collapsed due to termites—the causal explanation of the granary collapsing (termites) and the reason why people had been sitting under this particular granary (sun protection) would be considered to be independent from one another. For the Azande, however, “[w]itchcraft explains the coincidence of these two events” (Evans-Pritchard, 1937, p. 70).

Although magical thinking can be seen to be present at times in every human group, in “western” cultures it is considered as superstitious and is generally denigrated, as among the German students in our case. It is however construed as a legitimate cause for illness and certain other outcomes among the Yucatec Mayas, for instance. Results from our task show that intentionality alone was not a sufficient criterion for participants of any of the groups to attribute causality. Nonetheless, for the agency and counterfactual questions intentionality played a slightly more important role for the Yucatec, and even more for the Tseltal, than for the German and Mexican Spanish participants.

These results taken together imply that, although magical thinking can be taken to be a legitimate operating principle in certain cultures, it is not applicable to all domains or situations: it might be a legitimate sole cause to explain illness or death, but not in more everyday situations like the ones presented in our scenarios. In other words, thinking of an outcome is not always considered sufficient to determine causality, or more precisely, thinking is not always performative (Austin, 1975).

## Conclusion

Anthropologists as well as other social scientists often report that the way causality is inferred and interpreted is to some degree culturally shaped. Although it is not difficult to imagine how culture can influence the construal of causal relations between social actions and their effects (social causality), it is not always easy to demonstrate it through the collection of systematic data. Recent attempts to do so cross-culturally have shown that culture can influence attributions of causality even in sequences of physical events (physical causality; Bender and Beller, 2011a). Our cross-cultural study among four different groups is a comparable approach. Our results reveal a similar recognition of causality, showing that in all groups the Action-to-Outcome link was the most important for construing causality, more so than the Intention-to-Outcome or the Intention-to-Action links.

However, aside from these similarities there are very different interpretations cross-culturally of the relation between a cause and an outcome. What is striking from our study is the divergence in interpretation of exceptional (causal) relations across groups. While German and Mexican Spanish speakers have linguistic and cultural non-law-like concepts like *Zufall* or *casualidad* “chance, coincidence,” the two Mayan groups do not. In the Tseltal case, events are often seen as intermediary causes having “their own volition” *(y-oloj)* while Yucatec Mayas reject coincidences and attribute everything ultimately to “fate” *(sweerte)* and God's will. The interpretation of non-law-like relationship explanations and hence the distinction between causal relations and accidental sequences thus strongly depends on the cultural setting.

### Conflict of interest statement

The authors declare that the research was conducted in the absence of any commercial or financial relationships that could be construed as a potential conflict of interest.

## Footnotes

^1^This paper is a product of the ZiF project “The Cultural Constitution of Causal Cognition: Re-Integrating Anthropology into the Cognitive Sciences,” organized by Andrea Bender and Sieghard Beller. It was first presented at the Final Conference of this ZiF project on April 12, 2013. The authors' respective contributions are as follows: The project was initiated and the task design was initially proposed by Friedrich and Samland; the final design and cover stories were collectively created by the “Chance Group” of the ZiF project in which all the authors participated. The German data were collected and coded by Samland, the Tseltal data by Brown, and the Spanish Mexican and Yucatec data by Le Guen with the help of Ryan Taylor who ran the task among the Mexican students in Chiapas and Lorena Pool Balam who ran half of the Yucatec Mayas. Samland compiled the analyses for the four groups and did the statistical analyses. The initial conference presentation and the first draft of the paper were written by Le Guen. The revision of the paper was a joint effort again. We would like to thank the German, Mexican and Mayan participants, as well as the other members of the ‘Chance Group'. We thank Andrea Bender, Anita Schroven, and the fellows of the ZiF Research Group “The cultural constitution of causal cognition: Re-integrating anthropology into the cognitive sciences” (Bielefeld University, Germany) for inspiring discussions, and we thank the Max Planck Institute for Psycholinguistics in Nijmegen, The Netherlands, for fieldwork funding.

^2^We can cite studies like Choi et al. (1999), Morris and Peng (1994), or Morris et al. (1995), but these mainly focus on the eastern-western, individualism-collectivism distinction, which we will not address in this paper since we consider that these are quite arbitrary and would not apply in our study. We thank one of the reviewers for pointing out these lines of research to us.

^3^Throughout we use the terms “western” and “non-western” in quotation marks as shorthand for the more accurate WIERD term advocated by Henrich et al. (2010), to avoid its evaluative implications, although clearly some far-eastern and far southern cultural groups (e.g., Japanese, Australian, respectively) belong to the “western” category and many cultural groups—including our Mayan samples—situated in the western hemisphere belong to our “non-western” category.

^4^For the data collection in Germany and Mexico, an approval by an ethical board was not requested due to the small scale and non-critical nature of the study. The study was carried out in accordance with the ethical recommendations of the German Psychological Society (DGPs).

^5^Although scenario 8 could be considered as purely coincidental, it is also likely that, because of the interactional context of the task where participants are asked to judge scenarios, such a story triggers the assumption that some relevance must exist between the different events narrated. Indeed, the default assumption when someone tells a story is that it should have some relevance or some communicational intent (Grice, 1957). Consequently, we expected either that participants would see no causal link (i.e., consider it pure coincidence) or that they would be puzzled and perhaps invent some causal link not originally present in the story. In this latter case, we anticipated considerable inconsistency among participants of the same cultural group as well as between groups.

^6^Brown has been working for more than 40 years among the Tseltal Maya and Le Guen for almost 15 years among the Yucatec. Both are fluent in the local language (which they exclusively use for all interactions) and have been conducting ethnographic and linguistic studies as well as (psycho-) linguistic tasks for many years. Because of their experience (and the consulting of a native speaker in the case of Le Guen), possible cultural incongruities in the scenarios were avoided.

^7^The latter consider the criteria of “location,” but time and space are closely linked in our scenarios.

^8^We used the direct transitive formulation, e.g., “Did the hunter kill the deer?,” rather than the periphrastic formulation “Did the hunter cause the deer to die,” as the transitive is the least marked way to formulate this question about direct causation.

^9^Please note that in some cases two of the six cells were empty (no “maybe” responses). In these cases, a 2 × 2 contingency table was considered.

^10^Please note that in all chi-square tests, the N reported represents the number of data points of the comparison, and not the number of participants.

^11^We used Bonferroni-corrected *p*-values for the six single comparisons between the languages so that the chi-square results were considered to be significant if the corresponding *p*-value was lower than 0.008 in these cases.

^12^As proposed by one reviewer, we looked at the adjusted standardized residuals to determine which cells contributed most to the significant differences indicated by the conducted chi-square tests. Values higher than 2 or lower than −2 were considered to make a big contribution and the corresponding results are thus reported in the text. The tables with all adjusted standardized residuals are also provided in Appendix 2 of Supplementary Material.

^13^We are grateful to reviewer 2 for this interesting suggestion.

^14^This type of explanation was used for answers to some puzzling outcomes when the cover story implied a dream was under consideration. For instance, in scenario 4, the answer of one participant was that the man dreamed about the plates being broken just before they were actually broken, and woke up even though he had not heard them break. Precognition through dreams is an important principle, for it also explains why, for the Yucatec Mayas, things are not construed to just happen randomly and almost everything is ultimately attributed to “fate.”
