# Supplementary material for: Making sense of (exceptional) causal relations. A cross-cultural and cross-linguistic study
Source: Front Psychol. 2015 Oct 30;6:1645. doi: 10.3389/fpsyg.2015.01645 (PMC4626625; doi:10.3389/fpsyg.2015.01645)
Supplement: Supplementary file 1 [file DataSheet2.DOCX]

Appendix 1: Organization of scenarios and cover stories for the first 8 participants in the Yucatec, Tseltal and Mexican Spanish populations
(sc: scenario; part: participant; cs: cover story)

|  | Sc1 | Sc2 | Sc3 | Sc4 | Sc5 | Sc6 | Sc7 | Sc8 |
| --- | --- | --- | --- | --- | --- | --- | --- | --- |
| part1 | cs1 | cs2 | cs3 | cs4 | cs5 | cs6 | cs7 | cs8 |
| part2 | cs8 | cs1 | cs2 | cs3 | cs4 | cs5 | cs6 | cs7 |
| part3 | cs7 | cs8 | cs1 | cs2 | cs3 | cs4 | cs5 | cs6 |
| part4 | cs6 | cs7 | cs8 | cs1 | cs2 | cs3 | cs4 | cs5 |
| part5 | cs5 | cs6 | cs7 | cs8 | cs1 | cs2 | cs3 | cs4 |
| part6 | cs4 | cs5 | cs6 | cs7 | cs8 | cs1 | cs2 | cs3 |
| part7 | cs3 | cs4 | cs5 | cs6 | cs7 | cs8 | cs1 | cs2 |
| part8 | cs2 | cs3 | cs4 | cs5 | cs6 | cs7 | cs8 | cs1 |

Appendix 2: Contingency Tables for each question

Find below the contingency tables for all questions and the comparisons between all groups if each of the three links is present. The abbreviation A.S.R means ‘adjusted standardized residuals’ and the green-coloring emphasizes the cases in which the A.S.R is higher that 2 or lower than -2. As noted in footnote 11, values higher than 2 or lower than -2 were considered to make a big contribution and the corresponding results are thus reported in the text.

**Agency question 2: Did the agent cause the effect?**

1. **German-Tseltal Comparison**

| **A-O-link** |  | **no** | **yes** | **maybe** |  |
| --- | --- | --- | --- | --- | --- |
| German | Observed | 8 | 51 | 5 | 64 |
|  | Expected | 17,5 | 44 | 2,5 | 64 |
|  | A.S.R. | -3,8 | 2,7 | 2,3 |  |
| Tseltal | Observed | 27 | 37 | 0 | 64 |
|  | Expected | 17,5 | 44 | 2,5 | 64 |
|  | A.S.R. | 3,8 | -2,7 | -2,3 |  |
|  | Amount | 35 | 88 | 5 | 128 |

| **I-A-link** |  | **no** | **yes** | **maybe** |  |
| --- | --- | --- | --- | --- | --- |
| German | Observed | 27 | 26 | 11 | 64 |
|  | Expected | 29,5 | 29 | 5,5 | 64 |
|  | A.S.R. | -0,9 | -1,1 | 3,5 |  |
| Tseltal | Observed | 32 | 32 | 0 | 64 |
|  | Expected | 29,5 | 29 | 5,5 | 64 |
|  | A.S.R. | 0,9 | 1,1 | -3,5 |  |
|  | Amount | 59 | 58 | 11 | 128 |

| **I-O-link** |  | **no** | **yes** | **maybe** |  |
| --- | --- | --- | --- | --- | --- |
| German | Observed | 18 | 35 | 11 | 64 |
|  | Expected | 23,5 | 35,0 | 5,5 | 64 |
|  | A.S.R. | -2,0 | ,0 | 3,5 |  |
| Tseltal | Observed | 29 | 35 | 0 | 64 |
|  | Expected | 23,5 | 35,0 | 5,5 | 64 |
|  | A.S.R. | 2,0 | ,0 | -3,5 |  |
|  | Amount | 47 | 70 | 11 | 128 |

1. **German-Yucatec Comparison**

| **A-O-link** |  | **no** | **yes** | **maybe** |  |
| --- | --- | --- | --- | --- | --- |
| German | Observed | 8 | 51 | 5 | 64 |
|  | Expected | 7,5 | 54,0 | 2,5 | 64 |
|  | A.S.R. | ,3 | 1,5 | 2,3 |  |
| Yucatec | Observed | 7 | 57 | 0 | 64 |
|  | Expected | 7,5 | 54,0 | 2,5 | 64 |
|  | A.S.R. | -,3 | 1,5 | -2,3 |  |
|  | Amount | 15 | 108 | 5 | 128 |

| **I-A-link** |  | **no** | **yes** | **maybe** |  |
| --- | --- | --- | --- | --- | --- |
| German | Observed | 27 | 26 | 11 | 64 |
|  | Expected | 21,5 | 36,5 | 6,0 | 64 |
|  | A.S.R. | 2,1 | -3,7 | 3,0 |  |
| Yucatec | Observed | 16 | 47 | 1 | 64 |
|  | Expected | 21,5 | 36,5 | 6,0 | 64 |
|  | A.S.R. | -2,1 | 3,7 | -3,0 |  |
|  | Amount | 43 | 73 | 12 | 128 |

| **I-O-link** |  | **no** | **yes** | **maybe** |  |
| --- | --- | --- | --- | --- | --- |
| German | Observed | 18 | 35 | 11 | 64 |
|  | Expected | 19,0 | 39,0 | 6,0 | 64 |
|  | A.S.R. | -,4 | -1,4 | 3,0 |  |
| Yucatec | Observed | 20 | 43 | 1 | 64 |
|  | Expected | 19,0 | 39,0 | 6,0 | 64 |
|  | A.S.R. | ,4 | 1,4 | -3,0 |  |
|  | Amount | 38 | 78 | 12 | 128 |

1. **German-Mexican Spanish Comparison**

| **A-O-link** |  | **no** | **yes** | **maybe** |  |
| --- | --- | --- | --- | --- | --- |
| German | Observed | 8 | 51 | 5 | 64 |
|  | Expected | 14,0 | 46,7 | 3,3 | 64 |
|  | A.S.R. | -3,1 | 2,1 | 1,6 |  |
| Mexican | Observed | 13 | 19 | 0 | 32 |
|  | Expected | 7,0 | 23,3 | 1,7 | 32 |
|  | A.S.R. | 3,1 | -2,1 | -1,6 |  |
|  | Amount | 21 | 70 | 5 | 96 |

| **I-A-link** |  | **no** | **yes** | **maybe** |  |
| --- | --- | --- | --- | --- | --- |
| German | Observed | 27 | 26 | 11 | 64 |
|  | Expected | 30,0 | 25,3 | 8,7 | 64 |
|  | A.S.R. | -1,3 | ,3 | 1,5 |  |
| Mexican | Observed | 18 | 12 | 2 | 32 |
|  | Expected | 15,0 | 12,7 | 4,3 | 32 |
|  | A.S.R. | 1,3 | -,3 | -1,5 |  |
|  | Amount | 45 | 38 | 13 | 96 |

| **I-O-link** |  | **no** | **yes** | **maybe** |  |
| --- | --- | --- | --- | --- | --- |
| German | Observed | 18 | 35 | 11 | 64 |
|  | Expected | 22,7 | 33,3 | 8,0 | 64 |
|  | A.S.R. | -2,1 | ,7 | 2,0 |  |
| Mexican | Observed | 16 | 15 | 1 | 32 |
|  | Expected | 11,3 | 16,7 | 4,0 | 32 |
|  | A.S.R. | 2,1 | -,7 | -2,0 |  |
|  | Amount | 34 | 50 | 12 | 96 |

1. **Tseltal-Yucatec Comparison**

| **A-O-link** |  | **no** | **yes** | **maybe** |  |
| --- | --- | --- | --- | --- | --- |
| Tseltal | Observed | 27 | 37 |  | 64 |
|  | Expected | 17,0 | 47,0 |  | 64 |
|  | A.S.R. | 4,0 | -4,0 |  |  |
| Yucatec | Observed | 7 | 57 |  | 64 |
|  | Expected | 17,0 | 47,0 |  | 64 |
|  | A.S.R. | -4,0 | 4,0 |  |  |
|  | Amount | 34 | 94 |  | 128 |

| **I-A-link** |  | **no** | **yes** | **maybe** |  |
| --- | --- | --- | --- | --- | --- |
| Tseltal | Observed | 32 | 32 | 0 | 64 |
|  | Expected | 24,0 | 39,5 | ,5 | 64 |
|  | A.S.R. | 2,9 | -2,7 | -1,0 |  |
| Yucatec | Observed | 16 | 47 | 1 | 64 |
|  | Expected | 24,0 | 39,5 | ,5 | 64 |
|  | A.S.R. | -2,9 | 2,7 | 1,0 |  |
|  | Amount | 48 | 79 | 1 | 128 |

| **I-O-link** |  | **no** | **yes** | **maybe** |  |
| --- | --- | --- | --- | --- | --- |
| Tseltal | Observed | 29 | 35 | 0 | 64 |
|  | Expected | 24,5 | 39,0 | ,5 | 64 |
|  | A.S.R. | 1,6 | -1,4 | -1,0 |  |
| Yucatec | Observed | 20 | 43 | 1 | 64 |
|  | Expected | 24,5 | 39,0 | ,5 | 64 |
|  | A.S.R. | -1,6 | 1,4 | 1,0 |  |
|  | Amount | 49 | 78 | 1 | 128 |

1. **Tseltal-Mexican Spanish Comparison**

| **A-O-link** |  | **no** | **yes** | **maybe** |  |
| --- | --- | --- | --- | --- | --- |
| Tseltal | Observed | 27 | 37 |  | 64 |
|  | Expected | 26,7 | 37,3 |  | 64 |
|  | A.S.R. | ,1 | -,1 |  |  |
| Mexican | Observed | 13 | 19 |  | 32 |
|  | Expected | 13,3 | 18,7 |  | 32 |
|  | A.S.R. | -,1 | ,1 |  |  |
|  | Amount | 40 | 56 |  | 96 |

| **I-A-link** |  | **no** | **yes** | **maybe** |  |
| --- | --- | --- | --- | --- | --- |
| Tseltal | Observed | 32 | 32 | 0 | 64 |
|  | Expected | 33,3 | 29,3 | 1,3 | 64 |
|  | A.S.R. | -,6 | 1,2 | -2,0 |  |
| Mexican | Observed | 18 | 12 | 2 | 32 |
|  | Expected | 16,7 | 14,7 | ,7 | 32 |
|  | A.S.R. | ,6 | -1,2 | 2,0 |  |
|  | Amount | 50 | 44 | 2 | 96 |

| **I-O-link** |  | **no** | **yes** | **maybe** |  |
| --- | --- | --- | --- | --- | --- |
| Tseltal | Observed | 29 | 35 | 0 | 64 |
|  | Expected | 30,0 | 33,3 | ,7 | 64 |
|  | A.S.R. | -,4 | ,7 | -1,4 |  |
| Mexican | Observed | 16 | 15 | 1 | 32 |
|  | Expected | 15,0 | 16,7 | ,3 | 32 |
|  | A.S.R. | ,4 | -,7 | 1,4 |  |
|  | Amount | 45 | 50 | 1 | 96 |

1. **Yucatec-Mexican Spanish Comparison**

| **A-O-link** |  | **no** | **yes** | **maybe** |  |
| --- | --- | --- | --- | --- | --- |
| Yucatec | Observed | 7 | 57 |  | 64 |
|  | Expected | 13,3 | 50,7 |  | 64 |
|  | A.S.R. | -3,4 | 3,4 |  |  |
| Mexican | Observed | 13 | 19 |  | 32 |
|  | Expected | 6,7 | 25,3 |  | 32 |
|  | A.S.R. | 3,4 | -3,4 |  |  |
|  | Amount | 20 | 76 |  | 96 |

| **I-A-link** |  | **no** | **yes** | **maybe** |  |
| --- | --- | --- | --- | --- | --- |
| Yucatec | Observed | 16 | 47 | 1 | 64 |
|  | Expected | 22,7 | 39,3 | 2,0 | 64 |
|  | A.S.R. | -3,0 | 3,4 | -1,2 |  |
| Mexican | Observed | 18 | 12 | 2 | 32 |
|  | Expected | 11,3 | 19,7 | 1,0 | 32 |
|  | A.S.R. | 3,0 | -3,4 | 1,2 |  |
|  | Amount | 34 | 59 | 3 | 96 |

| **I-O-link** |  | **no** | **yes** | **maybe** |  |
| --- | --- | --- | --- | --- | --- |
| Yucatec | Observed | 20 | 43 | 1 | 64 |
|  | Expected | 24,0 | 38,7 | 1,3 | 64 |
|  | A.S.R. | -1,8 | 1,9 | -,5 |  |
| Mexican | Observed | 16 | 15 | 1 | 32 |
|  | Expected | 12,0 | 19,3 | ,7 | 32 |
|  | A.S.R. | 1,8 | -1,9 | ,5 |  |
|  | Amount | 36 | 58 | 2 | 96 |

**Counterfactual question: Would the effect have happened without the agent?**

1. **German-Tseltal Comparison**

| **A-O-link** |  | **no** | **yes** | **maybe** |  |
| --- | --- | --- | --- | --- | --- |
| German | Observed | 48 | 6 | 10 | 64 |
|  | Expected | 56,0 | 3,0 | 5,0 | 64 |
|  | A.S.R. | -4,3 | 2,5 | 3,3 |  |
| Tseltal | Observed | 64 | 0 | 0 | 64 |
|  | Expected | 56,0 | 3,0 | 5,0 | 64 |
|  | A.S.R. | 4,3 | -2,5 | -3,3 |  |
|  | Amount | 112 | 6 | 10 | 128 |

| **I-A-link** |  | **no** | **yes** | **maybe** |  |
| --- | --- | --- | --- | --- | --- |
| German | Observed | 28 | 17 | 19 | 64 |
|  | Expected | 42,5 | 10,0 | 11,5 | 64 |
|  | A.S.R. | -5,4 | 3,4 | 3,5 |  |
| Tseltal | Observed | 57 | 3 | 4 | 64 |
|  | Expected | 42,5 | 10,0 | 11,5 | 64 |
|  | A.S.R. | 5,4 | -3,4 | -3,5 |  |
|  | Amount | 85 | 20 | 23 | 128 |

| **I-O-link** |  | **no** | **yes** | **maybe** |  |
| --- | --- | --- | --- | --- | --- |
| German | Observed | 32 | 15 | 17 | 64 |
|  | Expected | 44,5 | 9,5 | 10,0 | 64 |
|  | A.S.R. | -4,8 | 2,7 | 3,4 |  |
| Tseltal | Observed | 57 | 4 | 3 | 64 |
|  | Expected | 44,5 | 9,5 | 10,0 | 64 |
|  | A.S.R. | 4,8 | -2,7 | -3,4 |  |
|  | Amount | 89 | 19 | 20 | 128 |

1. **German-Yucatec Comparison**

| **A-O-link** |  | **no** | **yes** | **maybe** |  |
| --- | --- | --- | --- | --- | --- |
| German | Observed | 48 | 6 | 10 | 64 |
|  | Expected | 51,0 | 8,0 | 5,0 | 64 |
|  | A.S.R. | -1,3 | -1,1 | 3,3 |  |
| Yucatec | Observed | 54 | 10 | 0 | 64 |
|  | Expected | 51,0 | 8,0 | 5,0 | 64 |
|  | A.S.R. | 1,3 | 1,1 | -3,3 |  |
|  | Amount | 102 | 16 | 10 | 128 |

| **I-A-link** |  | **no** | **yes** | **maybe** |  |
| --- | --- | --- | --- | --- | --- |
| German | Observed | 28 | 17 | 19 | 64 |
|  | Expected | 35,0 | 19,5 | 9,5 | 64 |
|  | A.S.R. | -2,5 | -1,0 | 4,7 |  |
| Yucatec | Observed | 42 | 22 | 0 | 64 |
|  | Expected | 35,0 | 19,5 | 9,5 | 64 |
|  | A.S.R. | 2,5 | 1,0 | -4,7 |  |
|  | Amount | 70 | 39 | 19 | 128 |

| **I-O-link** |  | **no** | **yes** | **maybe** |  |
| --- | --- | --- | --- | --- | --- |
| German | Observed | 32 | 15 | 17 | 64 |
|  | Expected | 34,0 | 21,5 | 8,5 | 64 |
|  | A.S.R. | -,7 | -2,4 | 4,4 |  |
| Yucatec | Observed | 36 | 28 | 0 | 64 |
|  | Expected | 34,0 | 21,5 | 8,5 | 64 |
|  | A.S.R. | ,7 | 2,4 | -4,4 |  |
|  | Amount | 68 | 43 | 17 | 128 |

1. **German-Mexican Spanish Comparison**

| **A-O-link** |  | **no** | **yes** | **maybe** |  |
| --- | --- | --- | --- | --- | --- |
| German | Observed | 48 | 6 | 10 | 64 |
|  | Expected | 46,7 | 6,0 | 11,3 | 64 |
|  | A.S.R. | ,6 | ,0 | -,8 |  |
| Mexican | Observed | 22 | 3 | 7 | 32 |
|  | Expected | 23,3 | 3,0 | 5,7 | 32 |
|  | A.S.R. | -,6 | ,0 | ,8 |  |
|  | Amount | 70 | 9 | 17 | 96 |

| **I-A-link** |  | **no** | **yes** | **maybe** |  |
| --- | --- | --- | --- | --- | --- |
| German | Observed | 28 | 17 | 19 | 64 |
|  | Expected | 28,0 | 16,7 | 19,3 | 64 |
|  | A.S.R. | ,0 | ,2 | -,2 |  |
| Mexican | Observed | 14 | 8 | 10 | 32 |
|  | Expected | 14,0 | 8,3 | 9,7 | 32 |
|  | A.S.R. | ,0 | -,2 | ,2 |  |
|  | Amount | 42 | 25 | 29 | 96 |

| **I-O-link** |  | **no** | **yes** | **maybe** |  |
| --- | --- | --- | --- | --- | --- |
| German | Observed | 32 | 15 | 17 | 64 |
|  | Expected | 34,7 | 13,3 | 16,0 | 64 |
|  | A.S.R. | -1,2 | ,9 | ,5 |  |
| Mexican | Observed | 20 | 5 | 7 | 32 |
|  | Expected | 17,3 | 6,7 | 8,0 | 32 |
|  | A.S.R. | 1,2 | -,9 | -,5 |  |
|  | Amount | 52 | 20 | 24 | 96 |

1. **Tseltal-Yucatec Comparison**

| **A-O-link** |  | **no** | **yes** | **maybe** |  |
| --- | --- | --- | --- | --- | --- |
| Tseltal | Observed | 64 | 0 |  | 64 |
|  | Expected | 59,0 | 5,0 |  | 64 |
|  | A.S.R. | 3,3 | -3,3 |  |  |
| Yucatec | Observed | 54 | 10 |  | 64 |
|  | Expected | 59,0 | 5,0 |  | 64 |
|  | A.S.R. | -3,3 | 3,3 |  |  |
|  | Amount | 118 | 10 |  | 128 |

| **I-A-link** |  | **no** | **yes** | **maybe** |  |
| --- | --- | --- | --- | --- | --- |
| Tseltal | Observed | 57 | 3 | 4 | 64 |
|  | Expected | 49,5 | 12,5 | 2,0 | 64 |
|  | A.S.R. | 3,2 | -4,2 | 2,0 |  |
| Yucatec | Observed | 42 | 22 | 0 | 64 |
|  | Expected | 49,5 | 12,5 | 2,0 | 64 |
|  | A.S.R. | -3,2 | 4,2 | 2,0 |  |
|  | Amount | 99 | 25 | 4 | 128 |

| **I-O-link** |  | **no** | **yes** | **maybe** |  |
| --- | --- | --- | --- | --- | --- |
| Tseltal | Observed | 57 | 4 | 3 | 64 |
|  | Expected | 46,5 | 16,0 | 1,5 | 64 |
|  | A.S.R. | 4,2 | -4,9 | 1,8 |  |
| Yucatec | Observed | 36 | 28 | 0 | 64 |
|  | Expected | 46,5 | 16,0 | 1,5 | 64 |
|  | A.S.R. | -4,2 | 4,9 | -1,8 |  |
|  | Amount | 93 | 32 | 3 | 128 |

1. **Tseltal-Mexican Spanish Comparison**

| **A-O-link** |  | **no** | **yes** | **maybe** |  |
| --- | --- | --- | --- | --- | --- |
| Tseltal | Observed | 64 | 0 | 0 | 64 |
|  | Expected | 57,3 | 2,0 | 4,7 | 64 |
|  | A.S.R. | 4,7 | -2,5 | -3,9 |  |
| Mexican | Observed | 22 | 3 | 7 | 32 |
|  | Expected | 28,7 | 1,0 | 2,3 | 32 |
|  | A.S.R. | -4,7 | 2,5 | 3,9 |  |
|  | Amount | 86 | 3 | 7 | 96 |

| **I-A-link** |  | **no** | **yes** | **maybe** |  |
| --- | --- | --- | --- | --- | --- |
| Tseltal | Observed | 57 | 3 | 4 | 64 |
|  | Expected | 47,3 | 7,3 | 9,3 | 64 |
|  | A.S.R. | 4,8 | -2,9 | -3,3 |  |
| Mexican | Observed | 14 | 8 | 10 | 32 |
|  | Expected | 23,7 | 3,7 | 4,7 | 32 |
|  | A.S.R. | -4,8 | 2,9 | 3,3 |  |
|  | Amount | 71 | 11 | 14 | 96 |

| **I-O-link** |  | **no** | **yes** | **maybe** |  |
| --- | --- | --- | --- | --- | --- |
| Tseltal | Observed | 57 | 4 | 3 | 64 |
|  | Expected | 51,3 | 6,0 | 6,7 | 64 |
|  | A.S.R. | 3,1 | -1,5 | -2,6 |  |
| Mexican | Observed | 20 | 5 | 7 | 32 |
|  | Expected | 25,7 | 3,0 | 3,3 | 32 |
|  | A.S.R. | -3,1 | 1,5 | 2,6 |  |
|  | Amount | 77 | 9 | 10 | 96 |

1. **Yucatec-Mexican Spanish Comparison**

| **A-O-link** |  | **no** | **yes** | **maybe** |  |
| --- | --- | --- | --- | --- | --- |
| Yucatec | Observed | 54 | 10 | 0 | 64 |
|  | Expected | 50,7 | 8,7 | 4,7 | 64 |
|  | A.S.R. | 1,8 | ,8 | -3,9 |  |
| Mexican | Observed | 22 | 3 | 7 | 32 |
|  | Expected | 25,3 | 4,3 | 2,3 | 32 |
|  | A.S.R. | -1,8 | -,8 | 3,9 |  |
|  | Amount | 76 | 13 | 7 | 96 |

| **I-A-link** |  | **no** | **yes** | **maybe** |  |
| --- | --- | --- | --- | --- | --- |
| Yucatec | Observed | 42 | 22 | 0 | 64 |
|  | Expected | 37,3 | 20,0 | 6,7 | 64 |
|  | A.S.R. | 2,0 | ,9 | -4,7 |  |
| Mexican | Observed | 14 | 8 | 10 | 32 |
|  | Expected | 18,7 | 10,0 | 3,3 | 32 |
|  | A.S.R. | -2,0 | -,9 | 4,7 |  |
|  | Amount | 56 | 30 | 10 | 96 |

| **I-O-link** |  | **no** | **yes** | **maybe** |  |
| --- | --- | --- | --- | --- | --- |
| Yucatec | Observed | 36 | 28 | 0 | 64 |
|  | Expected | 37,3 | 22,0 | 4,7 | 64 |
|  | A.S.R. | -,6 | 2,7 | -3,9 |  |
| Mexican | Observed | 20 | 5 | 7 | 32 |
|  | Expected | 18,7 | 11,0 | 2,3 | 32 |
|  | A.S.R. | ,6 | -2,7 | 3,9 |  |
|  | Amount | 56 | 33 | 7 | 96 |

**Temporal question: Why did the effect happen at that moment?**

1. **German-Tseltal Comparison**

| A-O-link |  | **causal -story based** | **causal -imposed** | **chance** | **fate** | **I don't know** | **miscellaneous** | |
| --- | --- | --- | --- | --- | --- | --- | --- | --- |
| German | Observed | 48 | 0 | 8 | 1 | 1 | 6 | 64 |
|  | Expected | 53,5 | 0,5 | 4,5 | 0,5 | 1,5 | 3,5 | 64 |
|  | A.S.R. | -2,6 | -1 | 2,4 | 1 | -0,6 | 1,9 |  |
| Tseltal | Observed | 59 | 1 | 1 | 0 | 2 | 1 | 64 |
|  | Expected | 53,5 | 0,5 | 4,5 | 0,5 | 1,5 | 3,5 | 64 |
|  | A.S.R. | 2,6 | 1 | -2,4 | -1 | 0,6 | -1,9 |  |
|  | Amount | 107 | 1 | 9 | 1 | 3 | 7 | 128 |
|  |  |  |  |  |  |  |  |  |
| I-A-link |  | **causal -story based** | **causal -imposed** | **chance** | **fate** | **I don't know** | **miscellaneous** | |
| German | Observed | 28 | 12 | 10 | 3 | 3 | 8 | 64 |
|  | Expected | 38,0 | 9,0 | 8,0 | 1,5 | 2,5 | 5,0 | 64 |
|  | A.S.R. | -3,6 | 1,5 | 1,1 | 1,8 | ,5 | 2,0 |  |
| Tseltal | Observed | 48 | 6 | 6 | 0 | 2 | 2 | 64 |
|  | Expected | 38,0 | 9,0 | 8,0 | 1,5 | 2,5 | 5,0 | 64 |
|  | A.S.R. | 3,6 | -1,5 | -1,1 | -1,8 | -,5 | -2,0 |  |
|  | Amount | 76 | 18 | 16 | 3 | 5 | 10 | 128 |
|  |  |  |  |  |  |  |  |  |
| I-O-link |  | **causal -story based** | **causal -imposed** | **chance** | **fate** | **I don't know** | **miscellaneous** | |
| German | Observed | 31 | 7 | 9 | 4 | 4 | 9 | 64 |
|  | Expected | 39,0 | 8,0 | 6,5 | 2,0 | 3,5 | 5,0 | 64 |
|  | A.S.R. | -2,9 | -,5 | 1,5 | 2,0 | ,4 | 2,6 |  |
| Tseltal | Observed | 47 | 9 | 4 | 0 | 3 | 1 | 64 |
|  | Expected | 39,0 | 8,0 | 6,5 | 2,0 | 3,5 | 5,0 | 64 |
|  | A.S.R. | 2,9 | ,5 | -1,5 | -2,0 | -,4 | -2,6 |  |
|  | Amount | 78 | 16 | 13 | 4 | 7 | 10 | 128 |
|  |  |  |  |  |  |  |  |  |

1. **German-Yucatec Comparison**

| A-O-link |  | **causal -story based** | **causal -imposed** | **chance** | **fate** | **I don't know** | **miscellaneous** | |
| --- | --- | --- | --- | --- | --- | --- | --- | --- |
| German | Observed | 48 | 0 | 8 | 1 | 1 | 6 | 64 |
|  | Expected | 45,5 | 2,5 | 5,0 | 4,5 | 1,0 | 5,5 | 64 |
|  | A.S.R. | 1,0 | -2,3 | 2,0 | -2,4 | ,0 | ,3 |  |
| Yucatec | Observed | 43 | 5 | 2 | 8 | 1 | 5 | 64 |
|  | Expected | 45,5 | 2,5 | 5,0 | 4,5 | 1,0 | 5,5 | 64 |
|  | A.S.R. | -1,0 | 2,3 | -2,0 | 2,4 | ,0 | -,3 |  |
|  | Amount | 91 | 5 | 10 | 9 | 2 | 11 | 128 |
|  |  |  |  |  |  |  |  |  |
| I-A-link |  | **causal -story based** | **causal -imposed** | **chance** | **fate** | **I don't know** | **miscellaneous** | |
| German | Observed | 28 | 12 | 10 | 3 | 3 | 8 | 64 |
|  | Expected | 28,5 | 13,5 | 5,5 | 6,5 | 3,0 | 7,0 | 64 |
|  | A.S.R. | -,2 | -,6 | 2,8 | -2,0 | ,0 | ,6 |  |
| Yucatec | Observed | 29 | 15 | 1 | 10 | 3 | 6 | 64 |
|  | Expected | 28,5 | 13,5 | 5,5 | 6,5 | 3,0 | 7,0 | 64 |
|  | A.S.R. | ,2 | ,6 | -2,8 | 2,0 | ,0 | -,6 |  |
|  | Amount | 57 | 27 | 11 | 13 | 6 | 14 | 128 |
|  |  |  |  |  |  |  |  |  |
| I-O-link |  | **causal -story based** | **causal -imposed** | **chance** | **fate** | **I don't know** | **miscellaneous** | |
| German | Observed | 31 | 7 | 9 | 4 | 4 | 9 | 64 |
|  | Expected | 30,5 | 11,0 | 4,5 | 8,5 | 2,5 | 7,0 | 64 |
|  | A.S.R. | ,2 | -1,9 | 3,1 | -2,3 | 1,4 | 1,1 |  |
| Yucatec | Observed | 30 | 15 | 0 | 13 | 1 | 5 | 64 |
|  | Expected | 30,5 | 11,0 | 4,5 | 8,5 | 2,5 | 7,0 | 64 |
|  | A.S.R. | -,2 | 1,9 | -3,1 | 2,3 | -1,4 | -1,1 |  |
|  | Amount | 61 | 22 | 9 | 17 | 5 | 14 | 128 |

1. **German-Mexican Spanish Comparison**

| A-O-link |  | **causal -story based** | **causal -imposed** | **chance** | **fate** | **I don't know** | **miscellaneous** | |
| --- | --- | --- | --- | --- | --- | --- | --- | --- |
| German | Observed | 48 | 0 | 8 | 1 | 1 | 6 | 64 |
|  | Expected | 48,0 | 2,7 | 6,7 | ,7 | ,7 | 5,3 | 64 |
|  | A.S.R. | ,0 | -2,9 | ,9 | ,7 | ,7 | ,5 |  |
| Mexican | Observed | 24 | 4 | 2 | 0 | 0 | 2 | 32 |
|  | Expected | 24,0 | 1,3 | 3,3 | ,3 | ,3 | 2,7 | 32 |
|  | A.S.R. | ,0 | 2,9 | -,9 | -,7 | -,7 | -,5 |  |
|  | Amount | 72 | 4 | 10 | 1 | 1 | 8 | 96 |
|  |  |  |  |  |  |  |  |  |
| I-A-link |  | **causal -story based** | **causal -imposed** | **chance** | **fate** | **I don't know** | **miscellaneous** | |
| German | Observed | 28 | 12 | 10 | 3 | 3 | 8 | 64 |
|  | Expected | 28,7 | 16,0 | 8,0 | 2,0 | 2,0 | 7,3 | 64 |
|  | A.S.R. | -,3 | -2,0 | 1,3 | 1,2 | 1,2 | ,5 |  |
| Mexican | Observed | 15 | 12 | 2 | 0 | 0 | 3 | 32 |
|  | Expected | 14,3 | 8,0 | 4,0 | 1,0 | 1,0 | 3,7 | 32 |
|  | A.S.R. | ,3 | 2,0 | -1,3 | -1,2 | -1,2 | -,5 |  |
|  | Amount | 43 | 24 | 12 | 3 | 3 | 11 | 96 |
|  |  |  |  |  |  |  |  |  |
| I-O-link |  | **causal -story based** | **causal -imposed** | **chance** | **fate** | **I don't know** | **miscellaneous** | |
| German | Observed | 31 | 7 | 9 | 4 | 4 | 9 | 64 |
|  | Expected | 31,3 | 10,7 | 8,0 | 2,7 | 2,7 | 8,7 | 64 |
|  | A.S.R. | -,1 | -2,1 | ,7 | 1,4 | 1,4 | ,2 |  |
| Mexican | Observed | 16 | 9 | 3 | 0 | 0 | 4 | 32 |
|  | Expected | 15,7 | 5,3 | 4,0 | 1,3 | 1,3 | 4,3 | 32 |
|  | A.S.R. | ,1 | 2,1 | -,7 | -1,4 | -1,4 | -,2 |  |
|  | Amount | 47 | 16 | 12 | 4 | 4 | 13 | 96 |

1. **Tseltal-Yucatec Comparison**

| A-O-link |  | **causal -story based** | **causal -imposed** | **chance** | **fate** | **I don't know** | **miscellaneous** | |
| --- | --- | --- | --- | --- | --- | --- | --- | --- |
| Tseltal | Observed | 59 | 1 | 1 | 0 | 2 | 1 | 64 |
|  | Expected | 51,0 | 3,0 | 1,5 | 4,0 | 1,5 | 3,0 | 64 |
|  | A.S.R. | 3,5 | -1,7 | -,6 | -2,9 | ,6 | -1,7 |  |
| Yucatec | Observed | 43 | 5 | 2 | 8 | 1 | 5 | 64 |
|  | Expected | 51,0 | 3,0 | 1,5 | 4,0 | 1,5 | 3,0 | 64 |
|  | A.S.R. | -3,5 | 1,7 | ,6 | 2,9 | -,6 | 1,7 |  |
|  | Amount | 102 | 6 | 3 | 8 | 3 | 6 | 128 |
|  |  |  |  |  |  |  |  |  |
| I-A-link |  | **causal -story based** | **causal -imposed** | **chance** | **fate** | **I don't know** | **miscellaneous** | |
| Tseltal | Observed | 48 | 6 | 6 | 0 | 2 | 2 | 64 |
|  | Expected | 38,5 | 10,5 | 3,5 | 5,0 | 2,5 | 4,0 | 64 |
|  | A.S.R. | 3,4 | -2,1 | 1,9 | -3,3 | -,5 | -1,5 |  |
| Yucatec | Observed | 29 | 15 | 1 | 10 | 3 | 6 | 64 |
|  | Expected | 38,5 | 10,5 | 3,5 | 5,0 | 2,5 | 4,0 | 64 |
|  | A.S.R. | -3,4 | 2,1 | -1,9 | 3,3 | ,5 | 1,5 |  |
|  | Amount | 77 | 21 | 7 | 10 | 5 | 8 | 128 |
|  |  |  |  |  |  |  |  |  |
| I-O-link |  | **causal -story based** | **causal -imposed** | **chance** | **fate** | **I don't know** | **miscellaneous** | |
| Tseltal | Observed | 47 | 9 | 4 | 0 | 3 | 1 | 64 |
|  | Expected | 38,5 | 12,0 | 2,0 | 6,5 | 2,0 | 3,0 | 64 |
|  | A.S.R. | 3,1 | -1,4 | 2,0 | -3,8 | 1,0 | -1,7 |  |
| Yucatec | Observed | 30 | 15 | 0 | 13 | 1 | 5 | 64 |
|  | Expected | 38,5 | 12,0 | 2,0 | 6,5 | 2,0 | 3,0 | 64 |
|  | A.S.R. | -3,1 | 1,4 | -2,0 | 3,8 | -1,0 | 1,7 |  |
|  | Amount | 77 | 24 | 4 | 13 | 4 | 6 | 128 |

1. **Tseltal-Mexican Spanish Comparison**

| A-O-link |  | **causal -story based** | **causal -imposed** | **chance** | **fate** | **I don't know** | **miscellaneous** | |
| --- | --- | --- | --- | --- | --- | --- | --- | --- |
| Tseltal | Observed | 59 | 1 | 1 |  | 2 | 1 | 64 |
|  | Expected | 55,3 | 3,3 | 2,0 |  | 1,3 | 2,0 | 64 |
|  | A.S.R. | 2,3 | -2,3 | -1,2 |  | 1,0 | -1,2 |  |
| Mexican | Observed | 24 | 4 | 2 |  | 0 | 2 | 32 |
|  | Expected | 27,7 | 1,7 | 1,0 |  | ,7 | 1,0 | 32 |
|  | A.S.R. | -2,3 | 2,3 | 1,2 |  | -1,0 | 1,2 |  |
|  | Amount | 83 | 5 | 3 |  | 2 | 3 | 96 |
|  |  |  |  |  |  |  |  |  |
| I-A-link |  | **causal -story based** | **causal -imposed** | **chance** | **fate** | **I don't know** | **miscellaneous** | |
| Tseltal | Observed | 48 | 6 | 6 |  | 2 | 2 | 64 |
|  | Expected | 42,0 | 12,0 | 5,3 |  | 1,3 | 3,3 | 64 |
|  | A.S.R. | 2,7 | -3,3 | ,5 |  | 1,0 | -1,3 |  |
| Mexican | Observed | 15 | 12 | 2 |  | 0 | 3 | 32 |
|  | Expected | 21,0 | 6,0 | 2,7 |  | ,7 | 1,7 | 32 |
|  | A.S.R. | -2,7 | 3,3 | -,5 |  | -1,0 | 1,3 |  |
|  | Amount | 63 | 18 | 8 |  | 2 | 5 | 96 |
|  |  |  |  |  |  |  |  |  |
| I-O-link |  | **causal -story based** | **causal -imposed** | **chance** | **fate** | **I don't know** | **miscellaneous** | |
| Tseltal | Observed | 47 | 9 | 4 |  | 3 | 1 | 64 |
|  | Expected | 42,0 | 12,0 | 4,7 |  | 2,0 | 3,3 | 64 |
|  | A.S.R. | 2,3 | -1,7 | -,6 |  | 1,2 | -2,3 |  |
| Mexican | Observed | 16 | 9 | 3 |  | 0 | 4 | 32 |
|  | Expected | 21,0 | 6,0 | 2,3 |  | 1,0 | 1,7 | 32 |
|  | A.S.R. | -2,3 | 1,7 | ,6 |  | -1,2 | 2,3 |  |
|  | Amount | 63 | 18 | 7 |  | 3 | 5 | 96 |

1. **Yucatec-Mexican Spanish Comparison**

| A-O-link |  | **causal -story based** | **causal -imposed** | **chance** | **fate** | **I don't know** | **miscellaneous** | |
| --- | --- | --- | --- | --- | --- | --- | --- | --- |
| Yucatec | Observed | 43 | 5 | 2 | 8 | 1 | 5 | 64 |
|  | Expected | 44,7 | 6,0 | 2,7 | 5,3 | ,7 | 4,7 | 64 |
|  | A.S.R. | -,8 | -,7 | -,7 | 2,1 | ,7 | ,3 |  |
| Mexican | Observed | 24 | 4 | 2 | 0 | 0 | 2 | 32 |
|  | Expected | 22,3 | 3,0 | 1,3 | 2,7 | ,3 | 2,3 | 32 |
|  | A.S.R. | ,8 | ,7 | ,7 | -2,1 | -,7 | -,3 |  |
|  | Amount | 67 | 9 | 4 | 8 | 1 | 7 | 96 |
|  |  |  |  |  |  |  |  |  |
| I-A-link |  | **causal -story based** | **causal -imposed** | **chance** | **fate** | **I don't know** | **miscellaneous** | |
| Yucatec | Observed | 29 | 15 | 1 | 10 | 3 | 6 | 64 |
|  | Expected | 29,3 | 18,0 | 2,0 | 6,7 | 2,0 | 6,0 | 64 |
|  | A.S.R. | -,1 | -1,4 | -1,2 | 2,4 | 1,2 | ,0 |  |
| Mexican | Observed | 15 | 12 | 2 | 0 | 0 | 3 | 32 |
|  | Expected | 14,7 | 9,0 | 1,0 | 3,3 | 1,0 | 3,0 | 32 |
|  | A.S.R. | ,1 | 1,4 | 1,2 | -2,4 | -1,2 | ,0 |  |
|  | Amount | 44 | 27 | 3 | 10 | 3 | 9 | 96 |
|  |  |  |  |  |  |  |  |  |
| I-O-link |  | **causal -story based** | **causal -imposed** | **chance** | **fate** | **I don't know** | **miscellaneous** | |
| Yucatec | Observed | 30 | 15 | 0 | 13 | 1 | 5 | 64 |
|  | Expected | 30,7 | 16,0 | 2,0 | 8,7 | ,7 | 6,0 | 64 |
|  | A.S.R. | -,3 | -,5 | -2,5 | 2,7 | ,7 | -,7 |  |
| Mexican | Observed | 16 | 9 | 3 | 0 | 0 | 4 | 32 |
|  | Expected | 15,3 | 8,0 | 1,0 | 4,3 | ,3 | 3,0 | 32 |
|  | A.S.R. | ,3 | ,5 | 2,5 | -2,7 | -,7 | ,7 |  |
|  | Amount | 46 | 24 | 3 | 13 | 1 | 9 | 96 |
